# SWATH-MS for discovery of early biomarkers of drug-induced cardiotoxicity using an animal doxorubicin model

**DOI:** 10.1186/s40959-026-00484-0

**Published:** 2026-05-13

**Authors:** Rohan Shotton, Amy Campbell, Ivona Baricevic-Jones, Rachel Reed, Janet Kelsall, Joanna Williams, Howard Mellor, Richard D. Unwin, Kim Linton

**Affiliations:** 1 Division of Cancer Sciences, School of Medical Sciences, Faculty of Biology, Medicine and Health, The University of Manchester, Manchester, England; 2 The Christie NHS Foundation Trust, Manchester Academic Health Science Centre, Manchester, M20 4BX England; 3 The Stoller Biomarker Discovery Centre, Division of Cancer Sciences, School of Medical Sciences, Faculty of Biology, Medicine and Health, The University of Manchester, Manchester, England

## Abstract

**Background:**

Clinical use of anthracyclines is limited by cumulative dose-dependent cardiotoxicity, though the mechanisms responsible for this are complex and incompletely understood. Existing biomarkers of anthracycline-related cardiotoxicity, such as troponin and NT-proBNP, lack sensitivity for the earliest stages of cardiotoxicity, therefore limiting scope to switch to alternative, safer treatment. As such, a reliable early biomarker of anthracycline-related cardiotoxicity remains a key unmet research need.

**Methods:**

We investigated the rat myocardial proteome to identify novel biomarkers of anthracycline-related cardiotoxicity warranting further investigation for routine clinical application. Six groups of 8 Han-Wistar rats were dosed weekly for 1–7 doses with either doxorubicin 1.25 mg/kg or saline control, followed by terminal bleeds. Sequential window acquisition of all theoretical mass spectra (SWATH-MS) was used to compare the rat myocardial proteome at serial timepoints and between doxorubicin- and saline-treated animals. A shortlist of candidate biomarkers was generated by identifying proteins with significant log_2_ fold changes at early/mid timepoint dysregulation, sustained to later timepoints. Pathway analysis for human ortholog proteins was undertaken using the Uniprot database and STRING.

**Results:**

Three proteins were differentially expressed at early, mid- and late timepoints (myosin light chain 3, desmin and cysteine-rich protein 2). A further 10 proteins were differentially expressed at mid- and late timepoints. Pathway analysis identified biological processes enriched within the myocardial proteome, predominantly related to muscle, metal ion, haemostasis, lipid and inflammation processes. A candidate biomarker shortlist of 34 proteins was derived for future study.

**Conclusion:**

We used a rat anthracycline dosing schedule to recapitulate the development of cardiotoxicity observed in humans. We describe a shortlist of 34 proteins with promise as candidate biomarkers of the earliest stages of anthracycline-related cardiotoxicity and summarise pathway analysis of functionally enriched biological processes within the rat myocardial proteome during anthracycline treatment. Further study is underway to explore these shortlisted proteins in rat plasma, with a human study planned to validate best-performing biomarkers.

**Supplementary Information:**

The online version contains supplementary material available at 10.1186/s40959-026-00484-0.

## Introduction

Anthracyclines are an important chemotherapy class with efficacy against a range of solid and haematological malignancies. Agents such as doxorubicin, daunorubicin, idarubicin and epirubicin play an important role in the curative, perioperative and palliative treatment of several malignancies. In addition to adverse effects which are common to most cytotoxic agents, anthracyclines are notable for the risk of cardiotoxicity, particularly at higher cumulative doses [1]. The mechanisms underlying anthracycline-related cardiotoxicity are complex and incompletely understood, with current mechanistic hypotheses revolving around oxidative and nitrosative stress, iron and calcium dysregulation, topoisomerase-II beta, endothelial dysfunction, inflammation and hypercoagulability and circulating microRNA disruption [2].

Derivation of alternative, anthracycline-free regimens for patients with cardiac comorbidity has been attempted, however the response and survival rates of these anthracycline-free regimens have not matched those in anthracycline-containing regimens, for example in diffuse large B cell lymphoma [3]. Due to the unmatched clinical effectiveness of these drugs, many strategies have been proposed to aid early diagnosis of incipient anthracycline-related cardiotoxicity, with a focus on circulating biomarkers for ease of clinical application. Cardiac troponin (cTn) or N-terminal pro-brain natriuretic peptide (NT-proBNP) prior to, during and after anthracycline-based chemotherapy are inconsistently recommended in practice guidelines with troponin being most promising [4–8]. While an abnormally elevated level is highly positively predictive, troponin elevations occur late during the pathological development of cardiotoxicity [9] and towards the end of planned chemotherapy, therefore limiting scope to switch to alternative, safer treatment. Furthermore, whilst instigation of cardioprotective agents can be effective within the first year of completing anthracycline chemotherapy, earlier intervention during chemotherapy may be more effective [10]. As such, a reliable early biomarker of anthracycline-related cardiotoxicity remains a key unmet research need.

Here, we used a rat model of anthracycline-induced cardiotoxicity, previously shown to recapitulate the human anthracycline cardiotoxicity process [9] to study drug-related changes in the myocardial proteome and identify novel biomarkers of anthracycline-related cardiotoxicity that warrant further investigation in peripheral blood for routine clinical application.

## Methods

Animal experiments were conducted at the AstraZeneca Drug Safety and Metabolism laboratories, as previously described [9], in accordance with directive 2010/63/EU of the European Parliament.

### Rat treatment and sample preparation

**Fig. 1:**
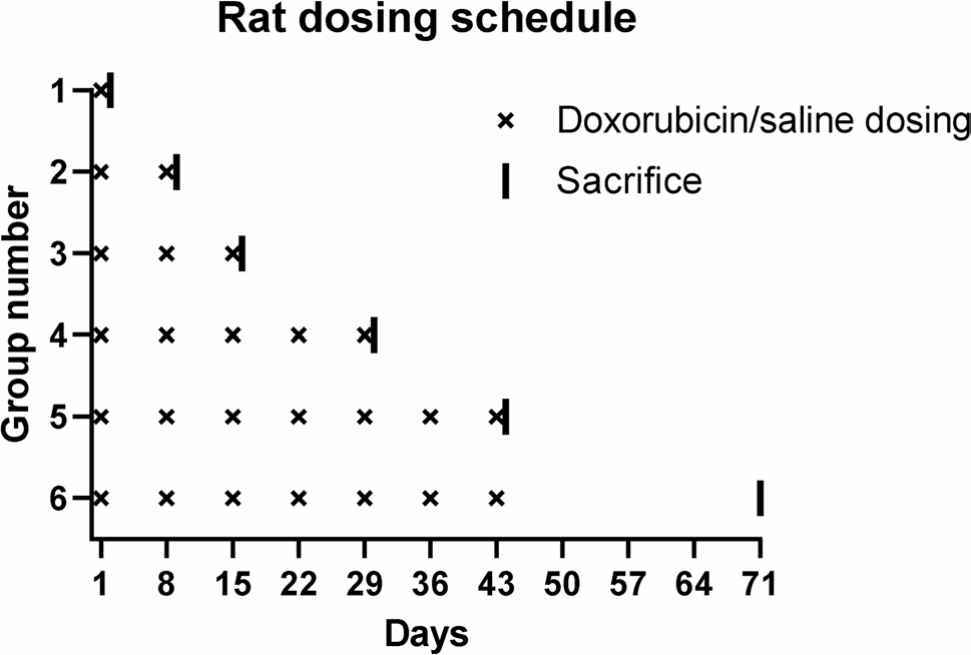
Rat dosing. Rats received doxorubicin 1.25 mg/kg or saline for 1, 2, 3, 5, 7 and 7 doses (groups 1–6 respectively; 96 rats in total), followed by culling on day 2, 9, 16, 30, 44 and 71 respectively

Han-Wistar rats were dosed weekly in groups of 8 with either intravenous doxorubicin 1.25 mg/kg or saline control, followed by terminal bleeds as illustrated in Fig. 1. Eight weeks of 1.25 mg/kg weekly dosing in rats has previously been shown to equate to maximum cumulative lifetime doxorubicin exposure in humans [9]. Rat myocardium lysate was prepared by homogenisation using a using a FASTPREP machine (Fisher Scientific). 30 mg of tissue lysate was added to 400µL of lysis/extraction buffer (1.5% sodium deoxycholate in 100mM ammonium bicarbonate) and homogenised for 40 s at 6.0 m/s, followed by incubation on ice for 5 min, for three repetitions. Samples were centrifuged at 14,000 x g for 5 min at 4 °C to pellet, and the supernatant transferred to a clean tube. Protein was quantified using a BCA assay.

### Spectral library generation and SWATH MS

Full details of methods are given in supplementary materials. In brief, pooled samples from each treatment group underwent liquid chromatography-mass spectrometry analysis using data-dependent acquisition (DDA) methods at both nano and micro-flow rates for peptide library generation. For differential expression analysis, sequential window acquisition of all theoretical mass spectra (SWATH-MS) [11] was used.

### Spectral library generation pipeline

DDA files generated via microflow LC on-line to mass spectrometry for 87 min (1 injection) and via nanoflow LC on-line to mass spectrometry for 135 min (1 injection) were searched against the UniProt SwissProt database on rat proteins. The peptide spectral matching was performed with X! tandem (Linux version 15.04.01.1). SpectraST was used to create a consensus spectral library after scoring by iProphet. Decoys were generated using the OpenSwathDecoyGenerator to allow the library to be used in openSWATH workflows [12].

### SWATH data analysis pipeline

SWATH maps were analysed against the bespoke rat heart spectral library. Quality control for each sample was performed via CVs computed between technical injection replicates. Samples were allowed through if median and 75% quantiles were 20% and 30% maximum respectively. Failed samples were re-analysed by SWATH-MS.

Feature alignment between SWATH maps was achieved via aligning multiple pyProphet files. Once SWATH maps were generated, processed and deemed satisfactory according to CV filtering, they were subjected to a pipeline to identify candidate biomarkers, including MSstats (normalisation across samples and transition to protein quantification), machine-learning to identify candidate biomarkers and RandomForest (run through multiple iterations; proteins that repeatedly occurred were retained and ranked ordered according to importance). For week-by-week differential protein expression between doxorubicin and saline-treated rats, significance (*p* ≤ 0.05) was determined by R package Limma using a Bayes approach.

### Candidate biomarker longlisting and shortlisting

From the full myocardial proteome, a longlist of candidate proteins was generated by filtering for proteins with statistically significant log_2_ fold changes either between dox- and saline-treated animals at two distinct timepoints, and at sequential timepoints for doxorubicin-treated rats. Significance *(p* ≤ 0.05*)* was determined by R package Limma (linear models for microarray data) using a Bayes approach. Timepoints were classified as Early (groups 1 and 2), Mid (groups 3 and 4) and Late (groups 5 and 6). Using a cut-off of log_2_ fold change ≥ 2, overall protein expression changes at Early, Mid and Late points were categorised as increased (I), decreased (D), stable (S) or indeterminate (?; applied in cases of unclear pattern of change). Proteins of interest for ongoing investigation were defined as those displaying a pattern of change in expression with significant early/mid-point log_2_fold change ≥ 2 that was sustained to the late time point.

To generate a candidate biomarker shortlist, proteins of interest with at least 2 peptides detected by mass spectrometry and implication in a functionally enriched pathway were selected. The shortlist was stratified into 3 prioritisation categories. Shortlisted proteins implicated in enriched pathways pertaining to the terms ‘heart’, ‘cardi*’, ‘muscle’, ‘myo*’ or ‘actin*’ were designated as Category 1. Category 2 consisted of the remaining shortlisted proteins plus manually identified extracellular matrix proteins (selected based on early/sustained change in expression). Category 3 was generated by further manual review of the longlist, prioritising proteins with significant log2 fold change, favourable pattern of change, higher number of detected peptides, higher number of predicted functional partners within the longlist, and implication in a greater number of functionally enriched pathways. Furthermore, proteins not detected in rat myocardium but which were predicted functional partners of a large number of longlisted proteins were also added to Category 3.

### Pathway analysis

Human ortholog proteins were identified via the Uniprot database [13]. Functional enrichments were reviewed using STRING [14] with the following settings: All active interaction sources (experiments, databases, co-expression, neighbourhood, gene fusion, co-occurrence) except textmining; minimum required interaction score ‘high’ (0.700); disconnected nodes hidden; Biological process OR KEGG pathways OR Wiki pathways OR reactome pathways.

## Results

### Protein identification and principal component analysis

**Fig. 2:**
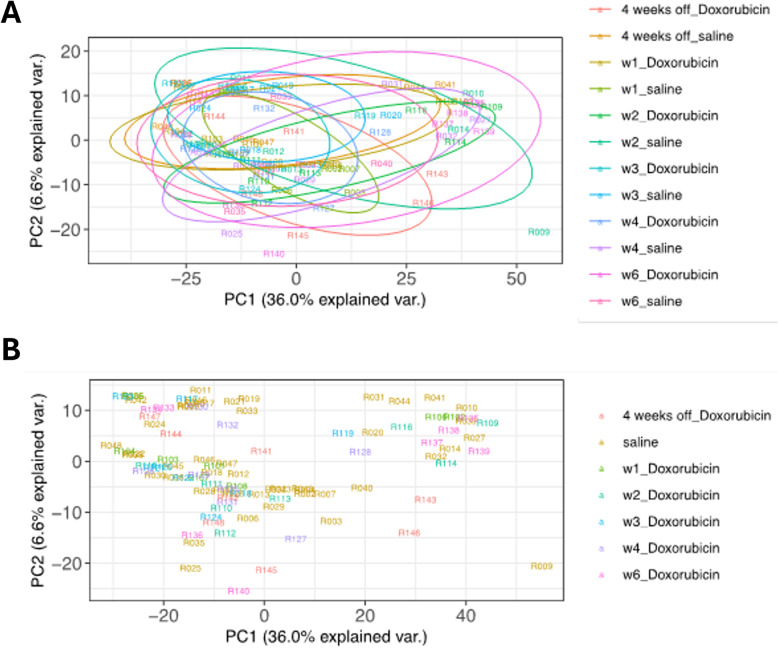
**a** and **b** Principal component analysis of all proteins quantified in at least 20% of samples (1317 proteins). **a** shows saline samples separately, and **b** shows saline samples grouped. PC1 and PC2 represent the first and second principal components, with 36.0% and 6.6% explained variance (explained var.) respectively. No clear separation was seen for any group

A spectral library was generated based on identification of 2318 proteins. SWATH-MS analysis identified 1489 proteins across all samples at a peptide false discovery rate of 1%. 1317 proteins were identified in at least 20% of samples (total 96 samples). Principal component analysis of this set revealed no clear separation of any group (Figs. 2a and b).

### Comparison of doxorubicin and saline-treated rats

#### Week-by-week differential protein expression between doxorubicin and saline-treated rats

**Fig. 3:**
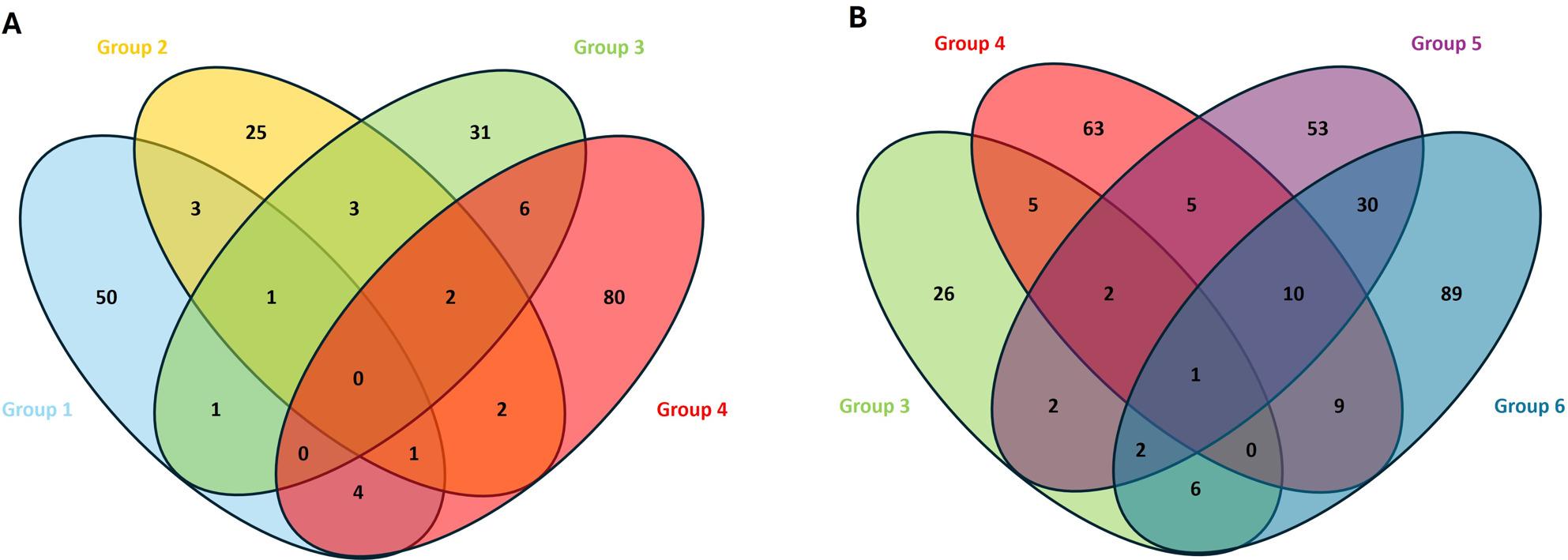
Numbers of proteins significantly differently expressed at each combination of timepoints; **a** between groups 1–4 (early vs. mid-phase); **b** between groups 3–6 (mid-phase vs. late)

Differential expression of 60, 37, 44, 95, 105 and 147 proteins between doxorubicin- and saline-treated rats was observed in groups 1–6, respectively. Protein differential expression is listed in supplementary Tables 2 and principal component analysis plots for each timepoint are shown in Supplementary Fig. 1a-f. Figure 3 illustrates the number of proteins that were significantly differentially expressed at each combination of timepoints.

#### Early and mid-phase protein expression changes

**Fig. 4:**
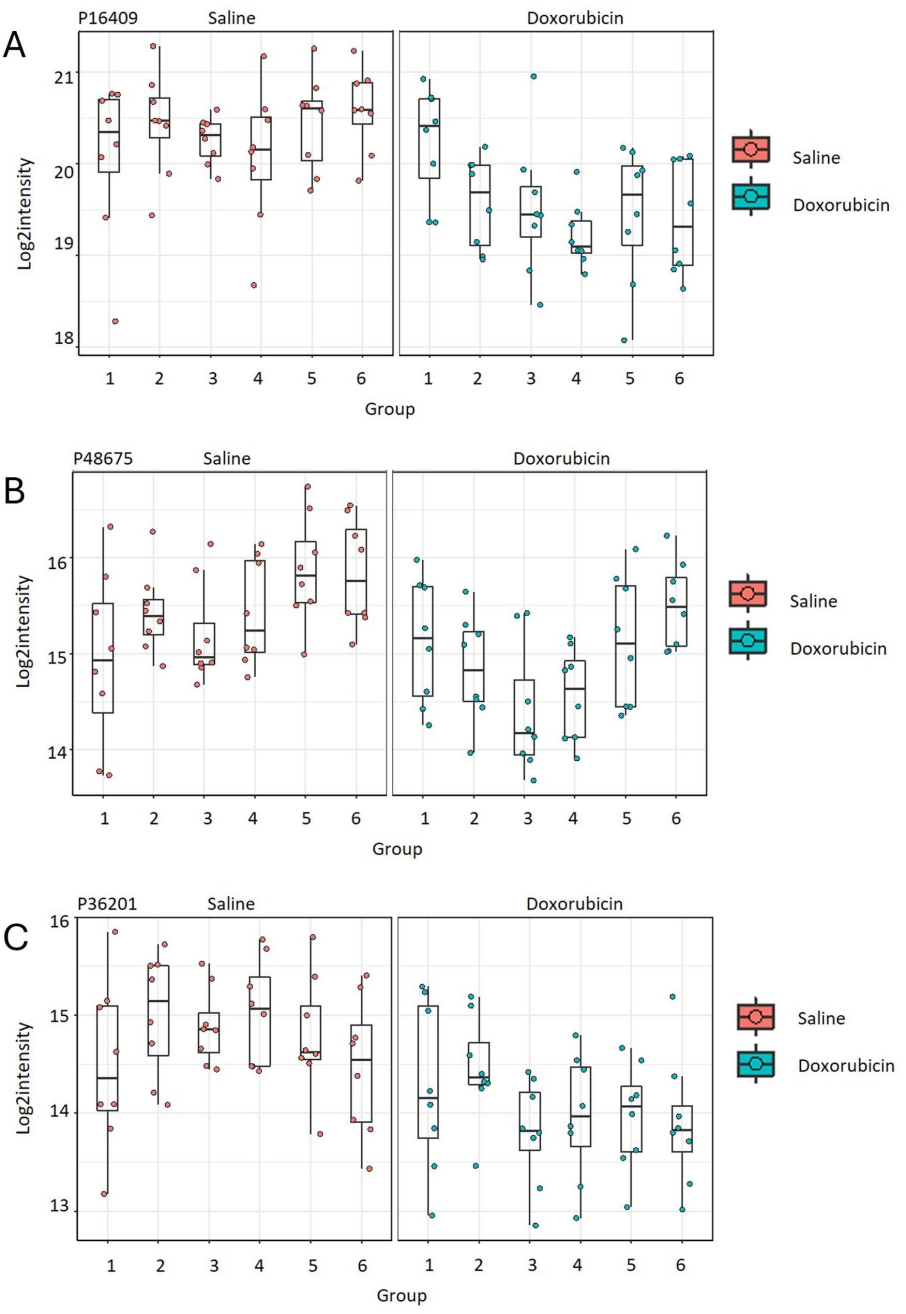
**a** (top): myosin light chain 3; downregulated after 2 weeks of doxorubicin compared to saline (group 2), then remains down at all time points. Group 2: log2FC -0.858, *p* = 0.00596; group 3: log2C -0.745, *p* = 0.0162; group 4: log2FC -0.862, *p* = 0.00931; group 5: log2FC -0.100, *p* = 0.00687; group 6: log2FC -1.180, *p* = 0.000523. **b** (centre): desmin; downregulated after 2 weeks of doxorubicin compared to saline (group 2), then remains down at all time points up to 7 weeks (group 5), before recovering at 7 weeks + 4 weeks off time point (Group 6) Group 2: log2FC -0.591, *p* = 0.0351; group 3: log2FC -0.790, *p* = 0.0171; group 4: log2FC -0.849, *p* = 0.00509; group 5: log2FC -0.747, *p* = 0.0309. **c** (bottom): cysteine-rich protein 2; downregulated after 3 weeks of doxorubicin (group 3) compared to saline, then remains down at all subsequent time points (but not significant at 7 weeks + 4 weeks off (group 6)). Group 3: log2FC -1.0838, *p* = 0.000344; group 4: log2FC -1.0695, *p* = 0.00206; group 5: log2FC -0.820, *p* = 0.0134

P16409 (myosin light chain 3) was significantly differentially expressed between doxorubicin and saline in groups 2, 3, 4, 5 and 6; P48675 (desmin) in groups 2, 3, 4 and 5; and P36201 (cysteine-rich protein 2) in groups 3, 4 and 5 (Fig. 4a-c).

Additionally, 10 proteins were differentially expressed in groups 4, 5 and 6, and 5 proteins in groups 4 and 5 alone, summarised in Table 1.

**Table 1:** Proteins significantly differentially expressed at multiple later time points

10 proteins significant in groups 4, 5 and 6											
Entry	Protein name	Gene name	Group 4			Group 5			Group 6		
			Log2FC	*p* value		Log2FC	*p* value		Log2FC		*p* value
P02563	Myosin-6 (Myosin heavy chain 6)	Myh6	-2.0469	0.0185		-1.792		0.00755	-2.457	0.00185	
P68035	Actin, alpha cardiac muscle 1	Actc1	-1.121	0.00135		-0.945		0.0188	-0.918	0.00282	
P20760	Ig gamma-2 A chain C region^a^	Igg-2a	-1.111	0.0116		-1.909		0.0193	-4.547	0.000000947	
P02564	Myosin-7 (Myosin heavy chain 7)	Myh7	-1.106	0.00200		-1.217		0.00442	-1.492	0.000202	
P63259	Actin, cytoplasmic 2	Actg1	-1.0973	0.00191		-0.940		0.00605	-0.921	0.00169	
P68136	Actin, alpha skeletal muscle	Acta1	-0.712	0.0106		-0.579		0.0434	-0.744	0.0123	
Q8K3K1	Usherin (Usher syndrome type IIa protein homolog)	Ush2a	-0.666	0.0225		-0.853		0.00556	-0.55	0.0222	
B0LPN4	Ryanodine receptor 2	Ryr2	-0.66094	0.0241		-1.348		0.0290	-1.221	0.000127	
P02600	Myosin light chain 1/3, skeletal muscle isoform	Myl1	-0.658	0.0220		-0.907		0.000562	-1.204	0.000888	
P70567	Tropomodulin-1	Tmod1	-0.602	0.00767		-0.627		0.0214	-0.573	0.0132	

5 proteins significant in groups 4 and 5 (but not 6)											
Entry	Protein name	Gene name	Log2FC		*p* value	Log2FC	*p* value				
Q63065	[Pyruvate dehydrogenase (acetyl-transferring)] kinase isozyme 1, mitochondrial	Pdk1	2.163		0.0423	3.852	0.000564				
Q8VHT6	Arsenite methyltransferase	As3mt	-2.116		0.00752	2.969	0.0262				
Q68FP1	Gelsolin (Actin-depolymerizing factor)	Gsn	-1.673		0.0135	-1.288	0.0355				
P63269	Actin, gamma-enteric smooth muscle (Alpha-actin-3)^b^	Actg2	-0.855		0.0209	-0.756	0.0139				
Q5XI32	F-actin-capping protein subunit beta (CapZ beta)	Capzb	-0.584		0.0113	-0.820	0.00899				

^a^ Igg-2a was also significantly downregulated in group 1 (log2FC -1.428, *p* = 0.0111)

^b^ Actg2 was also significantly downregulated in group 2 (log2FC -0.794, *p* = 0.0253)

Brain natriuretic peptide (BNP) and NT-proBNP were not detected; however, for atrial natriuretic peptide (P01161, ANP), there was a significant downregulation group 4 in doxorubicin-treated rats (log2FC-5.402, *p* = 0.00446; not measured in other groups).

#### Late protein expression changes

Myosin chains 6 (P02563) and 7 (P02564), and myosin light chain 1/3 (P02600) were persistently downregulated from 5 weeks of doxorubicin/saline (group 4). Ryanodine receptor 2 (BOLPN4) and tropomodulin-1 (P70567) showed similar patterns of change. There was no significant change in cardiac troponin abundance, with only a small log2 fold change of -0.21 at +4w for both cardiac troponin T (P50753) and troponin I (P23693) (*p* > 0.1).

### Comparison between sequential timepoints for doxorubicin-treated rats

In doxorubicin-treated rats, differential expression of 64, 137, 45, 102 and 47 proteins were observed between groups 1–6, respectively. Proteins with significant differential expression at each time point are displayed in supplementary Table 3.

### Candidate biomarkers

From a longlist of 144 candidate biomarkers (listed in Supplementary Table 1; derived as described in Methods), we identified 34 proteins of interest, including previously mentioned myosin-6, myosin-7, myosin light chain 3, desmin, Ig gamma-2 A chain C region, and gamma-enteric smooth muscle actin (summarised in Table 2).

**Table 2:** Thirty-four proteins demonstrating a favourable pattern of change in expression (defined as significant and sustained early/mid log 2fold change ≥ 2

D/D/D *N* = 6	I/I/I *N* = 2	I/S/I *N* = 3	D/S/D *N* = 3	D/D/S *N* = 4	S/D/D *N* = 12	S/I/I *N* = 4
Myosin-6	Major vault protein	AP-2 complex subunit alpha-2	Transthyretin	Dynamin-like 120 kDa protein, mitochondrial	Ryanodine receptor 2	Phosphoglucomutase-1
Myosin-7	Prothrombin	NEDD8-activating enzyme E1 regulatory subunit	Coagulation factor XIII A chain	Glutamine synthetase	Actin, alpha cardiac muscle 1	Protein phosphatase 1 regulatory subunit 12 A
Desmin		m-AAA protease-interacting protein 1, mitochondrial	Ig kappa chain C region, B allele	Syntaxin-7	Kynurenine–oxoglutarate transaminase 3	EH domain-containing protein 3
Myosin light chain 3				Histone H2A type 2-A	Gelsolin	Cystatin-C
Ig gamma-2 A chain C region					Tropomodulin-1	
Actin, gamma-enteric smooth muscle					Alpha-2-HS-glycoprotein	
					Actin, alpha skeletal muscle	
					Cysteine-rich protein 2	
					F-actin-capping protein subunit beta	
					Actin, cytoplasmic 2	
					Myosin light chain 1/3, skeletal muscle isoform	
					Usherin	

*D* decreased, *I* increased, *S* stable

Human orthologs were identifiable for each of the 34 proteins except Ig gamma-2 A chain C region and Ig kappa chain C region, B allele. For three of the proteins (usherin, histone H2A type 2-A and myosin light chain 1/3 skeletal muscle isoform), only a single peptide was detected via mass-spectrometry. Myosin light chain 1/3 skeletal muscle isoform was retained due to involvement in functionally enriched pathways, while usherin and histone H2A type 2-A were excluded in the absence of functionally enriched pathway involvement.

Pathway analysis, when filtered by Observed proteins ≥ 4 and Strength (Log10(observed / expected) ≥ 0.5 and False discovery rate *p* < 0.05, 186 enriched (overlapping) pathways remained, consisting of 129 unique proteins. The 186 enriched pathways were categorised as muscle processes (*n* = 44), metal ion processes (*n* = 16), haemostasis and angiogenesis processes (*n* = 14), lipid processes (*n* = 12), inflammation and immunity processes (*n* = 9), mitochondrial processes (*n* = 5), other metabolic processes (*n* = 20), and other (*n* = 67). These are summarised in Supplementary Table 4.

### Shortlist of candidate biomarkers

A shortlist of candidate biomarkers was derived, summarised in Table 3.

**Table 3:** Candidate biomarker shortlist

Protein name (gene name)		
Category 1 (*n* = 9)	Category 2 (*n* = 17)	Category 3 (*n* = 18)
Actin, alpha cardiac muscle 1 (*ACTC1*)	Alpha-2-HS-glycoprotein (AHSG)	Actin, aortic smooth muscle (ACTA2)**
Actin, alpha skeletal muscle (ACTA1)	Actin, gamma-enteric smooth muscle (ACTG2)	Actin, cytoplasmic 2 (ACTG1)
Desmin (DES)	All-trans-retinol 13,14-reductase	Alpha-actinin-2 (ACTN2)**
EH domain-containing protein 3 (EHD3)	AP-2 complex subunit alpha-2 (AP2A2)	Alpha-actinin-3 (ACTN3)**
F-actin-capping protein subunit beta (CAPZB)	Clusterin (CLU)*	Angiotensinogen (AGT)
Myosin light chain 3 (MYL3)	Coagulation factor XIII A chain (F13A1)	Apolipoprotein A-I (APOA1)
Myosin-7 (MYH7)	Dynamin-like 120 kDa protein, mitochondrial (OPA1)	Apolipoprotein A-II (APOA2)
Ryanodine receptor 2 (RYR2)	Glutamine synthetase (GLUL)	Apolipoprotein E (APOE)
Tropomodulin-1 (TMOD1)	Major vault protein (MVP)	Calcium/calmodulin-dependent protein kinase type II subunit delta (CAMK2D)
	Myosin-6 (MYH6)	Cystatin-C (CST3)
	NEDD8-activating enzyme E1 regulatory subunit (NAE1)	DNA-(apurinic or apyrimidinic site) endonuclease (APEX1)
	Plasminogen (PLG)*	Dystrophin (DMD)
	Protein phosphatase 1 regulatory subunit 12 A (PPP1R12A)	Gelsolin (GSN)
	Prothrombin (F2)	Myosin light chain 1/3, skeletal muscle isoform (MYL1)
	SPARC (SPARC)*	Myosin light chain 4 (MYL4)**
	Syntaxin-7 (STX7)	Myosin regulatory light chain 2 (MYL2)**
	Transthyretin (TTR)	Tropomyosin alpha-1 chain (TPM1)**
		Tropomyosin beta chain (TPM2)**

Category 1: Shortlisted proteins implicated in enriched pathways pertaining to the terms ‘heart’, ‘cardi*’, ‘muscle’, ‘myo*’ or ‘actin*’. Category 2: remaining shortlisted proteins plus manually identified extracellular matrix proteins (selected based on early/sustained change in expression). Category 3: further manually identified proteins as detailed in Methods

* Extracellular matrix protein; ** Protein not detected in rat myocardium but identified as a predicted functional partner of a significant number of longlisted human ortholog proteins

## Discussion

Anthracycline-related cardiotoxicity causes significant morbidity and mortality for cancer patients, and robust biomarkers signalling the earliest stages of cardiotoxicity are lacking. The currently available biomarkers troponin and NT-proBNP have limited utility for the early detection of incipient cardiotoxicity. Although recommended in clinical practice guidelines, these markers lack sensitivity for the earliest stages of cardiotoxicity, with elevations established to occur late in the pathological process [9], rendering treatment modification or instigation of cardioprotective strategies difficult.

Perturbations in the human and animal proteome offer significant potential for cardiotoxicity biomarker discovery and novel mechanistic insights. In this study we used an animal model of cardiotoxicity to generate a myocardial proteome from which candidate biomarkers of interest were identified for further study. Following dosing with a pre-defined schedule of doxorubicin or saline, we characterised differential protein expression in rat myocardium. While exploratory principal component analysis did not reveal any clear separation of any group, this may reflect other sources of variability rather than implying the absence of any treatment effect. A shortlist of candidate protein biomarkers was identified based on proteins with abundance demonstrating significant early/mid-point log_2_fold change ≥ 2 that was sustained to the late time point. In the current study, cardiac troponin, the leading biomarker recommended in guidelines, was detected in the myocardial proteome though with only a small, non-significant and late change in expression. This study reports candidate proteins with promise as early biomarkers of cardiotoxicity, warranting further study. Moreover, disruption of the rat myocardial proteome after anthracycline dosing here identifies the relevance of key groups of functionally enriched biological processes, as discussed below.

### Muscle pathways

In doxorubicin-treated rats, we report downregulation of several proteins relating to the sarcomere including myosin and actin isoforms, desmin and tropomodulin. A number of key biological process pathways were enriched within the protein longlist, including many pertaining to striated/cardiac muscle development and function and regulation and sarcomere organisation. Notably, cardiac muscle cell apoptosis (strength 1.21) was also enriched, and all 9 longlisted proteins implicated in the dilated cardiomyopathy pathway (strength 1.13) were also included in the shortlist (ACTC1, MYH7, DMD, RYR2, AGT, DES, MYL3, MYH6, ACTG1).

### Myosin isoforms

We report downregulation of myosin light chain 3 at all time points after doxorubicin dosing compared to saline, as well as downregulation of myosin-6 and − 7 at later timepoints. Several studies have reported associations between myosin light chain 3 dysregulation and cardiotoxicity. In our study, myosin light chain 3 was the most promising candidate biomarker, with persistent downregulation seen after just 2 weeks of doxorubicin (group 2). A previous study reported disruptions in the proteome of rabbits dosed with a heart-failure inducing weekly daunorubicin (3 mg/kg) schedule [15]. In keeping with our observations, marked downregulation of myosin light chain isoforms 1 and 2 was observed. A mouse study of doxorubicin also reported downregulation of both myosin light chain 3 and cardiac troponin I from 1 week to 6 weeks after the 5th dose with continued progression of cardiac disease [16]. Interestingly, the extent of acute phase rise of myosin light chain 3 (prior to subsequent downregulation) predicted the severity of chronic cardiotoxicity better than troponin, particularly in males. The authors infer that an initial acute phase rise in troponin and myosin light chain 3 occurred prior to downregulation, which may be a consequence of the dosing schedule used (5 mg/kg for mice vs. 1.25 mg/kg for rats in our study). In another rat study involving a single doxorubicin dose of 40 mg/kg, myosin light chain 3 upregulation showed superior receiver operator characteristic area under curve (ROC-AUC 0.916) for cardiac or musculoskeletal injury versus the more conventional biomarkers aspartate aminotransferase, lactate dehydrogenase and creatine kinase [17].

Myosin light and heavy chains were also dysregulated in a study of human pluripotent stem cell-derived cardiomyocytes following doxorubicin exposure [18]. Additionally, the 199 differentially expressed proteins notably included troponins, tropomyosins, and ribosomal and mitochondrial-related proteins. Similarly to our study, pathway analysis identified over-representation of cardiac muscle contraction, hypertrophic cardiomyopathy, dilated cardiomyopathy and ribosome pathways at all time points following doxorubicin exposure. Each of these pathways except ribosome pathways was functionally enriched in our dataset. Proteomic data were subsequently integrated with prior transcriptomic findings reported by the same group [19, 20]. Significant overlap was identified between differentially expressed proteins and messenger RNA (mRNA)/microRNAs in changes including downregulation of cardiomyocyte myofibrillar genes/proteins at early time points following doxorubicin exposure, upregulation of mitochondrial function genes/proteins, and dysregulation of various ion channels and ribosome pathways.

### Actin and related proteins

We report initial stability followed by persistent downregulation of the actin isoforms alpha cardiac muscle 1, alpha skeletal muscle, and cytoplasmic-2 at groups 4–6 in doxorubicin- versus saline-treated rats. Gamma-enteric smooth muscle actin was downregulated in groups 2–5. Additionally, the actin capping proteins F-actin-capping protein subunit beta and gelsolin were both significantly downregulated in groups 4 and 5. These findings recapitulate those of older in vitro studies, which reported dose-dependent downregulation of both actin and alpha cardiac actin mRNA in doxorubicin-treated cultured cardiomyocytes [21, 22]. In a human study, plasma gelsolin concentration was reduced immediately after first anthracycline exposure for breast cancer [23].

### Desmin

In the myocardium of doxorubicin-treated rats compared to those treated with saline, we observed downregulation of the Z-disc associated protein desmin after 2 weeks of doxorubicin (group 2), with downregulation persisting until recovery 4 weeks after a 7th dose (group 6). In an in vitro study, rat cardiomyocytes were exposed to an apoptosis-inducing dose of anthracycline, with subsequent protein profiles compared using matrix assisted laser desorption ionization time-of-flight (MALDI-TOF/TOF) mass spectrometry [24]. Desmin was upregulated compared to controls, contrary to our findings. Similar myocardial desmin upregulation was also reported in the rabbit study by Štěrba et al. [15]. On immunohistochemical confirmation, the source of increased desmin was identified solely as cardiomyocytes, with desmin disorganisation correlating with the degree of left ventricular dysfunction. Cardiomyocytes with mild morphological damage demonstrated a moderate increase in desmin abundance though with preserved intracellular localisation, while severe morphological damage was associated with substantially upregulated desmin and loss of intracellular organisation. The authors thus propose desmin as a potential early marker of anthracycline cardiotoxicity. The contradictory downregulation of desmin identified in our study again likely reflects differences in dosing schedules between an apoptosis-inducing in-vitro doses and the lower, chronic dosing model used in our study (equivalent to that which causes cardiotoxicity in humans).

### Metal ion processes

Enriched pathways pertaining to metal ion processes chiefly related to sodium or calcium ion transport. In particular, we observed enrichment of pathways implicating the shortlisted proteins ryanodine receptor 2, dystrophin and calcium/calmodulin-dependent protein kinase type II subunit delta.

We observed initial stability of ryanodine receptor 2, followed by significant decreases in doxorubicin-treated rats in groups 4–6, following a significant drop from groups 2 to 3. Yuan et al. also report downregulation of the same protein in rat plasma via an integrated proteomic/metabolomic approach, in which rats were dosed with intraperitoneal saline or doxorubicin 3 mg/kg every 7 days for 6 doses [25]. Decreased expression or deficiency of ryanodine receptor 2 has been associated with calcium leak from sarcoplasmic reticulum, impairing cardiac contraction and in turn leading to impaired mitochondrial ATP production [26].

Calcium/calmodulin-dependent protein kinase type II subunit delta (CaMK2d) was transiently downregulated in doxorubicin-treated hearts in group 3, followed by late recovery (supplementary Table 1). CaMK2d activation by receptor-interacting protein 3 (RIP3)-induced phosphorylation or oxidation has been implicated in the pathogenesis of heart failure and myocardial infarction via induction of apoptosis, inflammation and necroptosis [27, 28]. In mice, CaMK2d activation by doxorubicin exposure has been demonstrated to induce calcium leak from sarcoplasmic reticulum [29]. Both of these processes were ameliorated in RIP3-deficient or CaMK2d-knockout rats/mice respectively. Additionally, CaMK2d mRNA has been shown to be downregulated in rats which develop doxorubicin-cardiomyopathy [30].

We observed initial stability followed by mid- and late-phase upregulation of endosome-based Eps15 homology domain 3 (EHD3; upregulated from groups 2 to 3 and groups 5 to 6). These findings are in keeping with those of Gudmundsson et al., who reported EHD3 upregulation in both a rat chronic heart failure model and a human non-ischaemic heart failure model [31]. Reactive oxygen species exposure was observed to result in EHD3 overexpression, in turn leading to downstream overexpression of the sodium/calcium exchange protein NCX1. Conversely, however, Curran et al. observed in a mouse model that EHD3 deficiency was associated with structural and functional heart defects [32].

### Haemostasis and angiogenesis

Disruption of normal haemostasis has been reported as a contributory mechanism for anthracycline cardiotoxicity due to activation of the clotting cascade [33] following doxorubicin exposure. Key enriched pathways included those pertaining to platelet activation and the clotting cascade. We observed early and late upregulation of prothrombin (factor 2) from groups 1–2 and 4–6. Grover at al also observed acute upregulation of prothrombin in mice treated with doxorubicin, with data suggesting that downstream activation of protease-activated receptor 1 (PAR1) contributes to cardiotoxicity [34]. Prothrombin is converted to thrombin, one of the roles of which is to activate factor 13, contributing to clot stabilisation [35]. We observed downregulation of factor 13 A1 from groups 1 to 2 and 5 to 6. We also report disruption of plasminogen, with an initial rise from groups 1 to 2 followed by later downregulation from groups 4 to 5 and 5 to 6. Doxorubicin is known to induce plasminogen activator inhibitor 1 (PAI-1), which ordinarily inhibits plasminogen activation to plasmin, thereby inhibiting fibrinolysis. In cultured cardiomyocytes, Ghosh et al. observed that a PAI-1 inhibitor prevented doxorubicin-induced senescence [36]. Our findings confirm that doxorubicin causes complex disruption of platelet function, the clotting cascade and angiogenesis, all of which may contribute to cardiotoxicity.

### Lipid processes

We observed late-phase upregulation in apolipoproteins A1, A2 and E, none of which have been investigated as biomarkers of anthracycline cardiotoxicity. We report upregulation of apolipoprotein A-I at later time points after early and mid-point stability. Upregulation was also reported for apolipoprotein A-I precursor in myocardium from rats treated with a single dose of doxorubicin 2.5 mg/kg versus saline [37]. In addition to apolipoprotein A-I precursor, MALDI-TOF mass spectrometry identified dysregulation of a large number of proteins, including upregulation of actin and troponin T2. Similarly, in the study described above by Bao et al., apolipoprotein A-I was also upregulated [24]. Apolipoprotein A1 has been shown to be protective against anthracycline cardiotoxicity [38, 39], and increased serum apolipoprotein A2 levels have been associated with a protective effect against future cardiovascular events in patients undergoing percutaneous coronary intervention [40]. Both were implicated in a number of enriched pathways. Of note, the two most strongly enriched pathways from the protein longlist were Positive regulation of cholesterol esterification (strength 1.8; APOA1, APOE, AGT and APOA2) and high-density lipoprotein particle clearance (strength 1.75; APOA1, APOE, APOA2, HDLBP).

### Other dysregulated proteins

We observed early and late downregulation of transthyretin, with implication in several pathways pertaining to inflammation/immunity processes. Similar findings were reported by Yuan et al. [25], associated with significant dysregulation in pathways including glutathione metabolism, cardiac muscle contraction, fructose and mannose metabolism, glycolysis and gluconeogenesis, complement and coagulation pathways. Of the 278 differentially expressed proteins between control and doxorubicin groups, 25 were closely related to differential metabolite profiles, including downregulation of transthyretin and ryanodine receptor 2, as seen in our data.

Protein dysregulation different to that reported in our study has been reported in other studies, though these are often small in size. Nguyen et al. describe a tandem in vitro study of human-derived cardiomyocytes and cardiac biopsies from 8 patients with anthracycline-related heart failure, reporting the proteomic changes at 7 time-points during and following anthracycline exposure via mass spectrometry [41]. Four nodal ‘hub’ and 7 other significant ‘high module membership’ proteins were reported, none of which are reported in our study. The 4 hub proteins were Cullin-associated NEDD8-dissociated protein 1, Endoplasmic reticulum chaperone BiP, heat shock protein beta-1, BAG family molecular chaperone regulator 3, and the 7 high module membership proteins were SH3 domain-binding glutamic acid-rich-like protein 3, Elongation factor 1-delta, mitochondrial import inner membrane translocase subunit Tim13, mitochondrial ATP synthase subunit beta, mitochondrial 2,4-dienoyl-CoA reductase, and mitochondrial-processing peptidase subunit beta. In a mouse study, a slow off-rate modified aptamer (SOMAmer) multiplex platform was used to characterise protein abundance changes during/following a schedule of doxorubicin versus saline treatment [42]. Eighteen proteins were identified as over-expressed by fold-change ≥ 1.2. Of these, an early increase prior to increase of cardiac troponin I was reported for 6 proteins, which remained elevated at higher cumulative doses of doxorubicin (NOTCH1, von Willebrand factor, mitochondrial glutamate carrier 2, Wnt inhibitor factor 1, legumain and mannan-binding lectin serine protease 1). Pre-treatment with dexrazoxane limited the rise in two of these (NOTCH1 and von Willebrand factor), suggesting a promising role for these proteins as predictive biomarkers for ARC.

In humans, Beer and colleagues used a liquid chromatography-mass spectrometry approach to proteomic biomarker discovery on plasma samples in a cohort of 3 patients with cardiac dysfunction following treatment with doxorubicin and/or trastuzumab for breast cancer [43]. The greatest difference between patients with and without cardiac dysfunction was identified for immunoglobulin E (IgE), with significantly lower levels at baseline and all subsequent timepoints in patients without cardiac dysfunction. These findings suggest that as well as implicating the immune system in the pathogenesis of cardiotoxicity, baseline IgE levels may be a useful predictive biomarker. An alternative platform was used by Mörth et al. in a human study of cardiotoxicity in 95 patients treated for diffuse large B cell lymphoma, using the OLINK proximity extension immunoassay [44]. In this cohort of patients, compared to healthy controls, the proteins Spondin-1 and Interleukin-1 receptor type 1 were reported as correlating with pre-treatment and emergent cardiovascular disease respectively.

Our findings both confirm established findings from other studies detailed above, and extend existing knowledge in several important ways. Consistent with other studies, we report dysregulation of sarcomeric proteins and pathways, supporting prior findings in anthracycline cardiotoxicity relating to myofibrillar disruption and abnormalities of excitation-contraction coupling. Our observation of key functionally enriched pathways pertaining to haemostasis, coagulation and lipid metabolism also align with findings reported previously, and mechanistic insights from this study reinforce findings described in the literature. In contrast to many previous studies, however, our study uses a longitudinal approach to cardiotoxicity biomarker discovery, using the myocardial proteome in a clinically relevant animal model as the foundation of future studies in peripheral blood. SWATH-MS enabled reliable protein quantification in serial timepoints, facilitating selection of proteins with early or mid-phase dysregulation which was sustained to later stages. It is acknowledged that a limitation of this study is the lack of orthogonal validation of our findings via alternative methods (for example Western blotting), which was not feasible due to unavailability of original biological samples. To mitigate this, we applied stringent false discovery rate control to pathway analysis and prioritised proteins with multiple unique peptides detected (detailed in Supplementary Table 1).

## Conclusion

In summary, we describe proteomic changes identified in rat myocardium following a schedule of human-equivalent doxorubicin dosing. By comparing week-to-week changes in doxorubicin-treated rats and also week-by-week differences between doxorubicin- and saline-treated rats, we report the kinetics of changes in key proteins which may have a role as predictive biomarkers of anthracycline-related cardiotoxicity. In a further study planned by our group, we aim to validate these myocardial proteins in rat plasma, using enzyme-linked immunosorbent assay. Best-performing biomarkers will then be evaluated in a translational clinical study, correlating novel biomarker changes with cardiac MRI findings before and after chemotherapy.

## Supplementary Information

Supplementary Material 1. [11, 45, 46].

## Data Availability

Requests for source data will be considered upon request to the chief investigator, Dr Kim Linton, via email.
