# Supplementary material for: SWATH-MS for discovery of early biomarkers of drug-induced cardiotoxicity using an animal doxorubicin model
Source: Cardiooncology. 2026 May 13;12:87. doi: 10.1186/s40959-026-00484-0 (PMC13343697; doi:10.1186/s40959-026-00484-0)
Supplement: Supplementary file 1 — Supplementary Material 1. [11, 45, 46]. [file 40959_2026_484_MOESM1_ESM.docx]

# **Supplementary material**

## Spectral library generation and SWATH-MS methods

One hundred micrograms of protein from each of the 0.9% saline group and doxorubicin-treated group were pooled, using equal contribution from individual animals. Pooled samples were reduced for 2 minutes at 95°C in the presence of 5mM dithiothreitol. Once cooled to room temperature, samples were alkylated with iodoacetamide at a final 50mM concentration in a final volume of 220uL.Samples were loaded into 5 replicate wells and gel fractionated by SDS PAGE. The gel was stained with Coomassie Instant Blue, and the gel lane cut into 50 bands using a scalpel. Gel pieces were de-stained and vacuum dried, followed by overnight in-gel tryptic digestion in rehydrated samples as follows: Dried gel pieces were rehydrated by immersing in 100 µL 50mM ammonium bicarbonate (ABC) /10% acetonitrile (ACN) containing 5 ng/µL trypsin, and the multiwell plate was sealed and centrifuged at 1000g for 1 minute at 4°C. The gel pieces were stored on ice for 30 minutes, with 100 µL 50mM ABC added to each sample well after 15 minutes to ensure the gel pieces were fully immersed, followed by overnight incubation at 37°C on a shaking platform. Following digestion, 20 µL 10% (v/v) formic acid (FA) was added to each sample and an equal volume (220 µL) 100% acetonitrile was added to each sample well and the plate shaken gently for 20 minutes at room temperature. Supernatant from the 5 replicate bands were pooled across each fraction and transferred to ten 2mL Non Lo-Bind Eppendorf tubes labelled Fraction 1 to 10. Remaining peptides were extracted from the gel by adding 200µL 5% (v/v) FA/50% (v/v) ACN to the remaining gel pieces and shaken for a further 20 minutes. The supernatant was removed and combined with the relevant previous fraction, and a second identical extraction was performed. The peptides were vacuum dried for ~ 4 hours at 55°C in a centrifugal evaporator and stored at -20oC until ready for LC-MS analysis.

Liquid chromatography-mass spectrometry was performed at both nano and microlow rates for library generation. Peptides were separated on a NanoLC™ 425 System (Eksigent) operating in trap-elute mode. Mobile phase A was 0.1% (v/v) Formic acid/LC-MS grade water and Mobile phase B was 0.1% (v/v) Formic acid/Acetonitrile. For nanoflow analysis, the LC was set up with a PicoFrit® column- PF7510-250H354-3P C18 Reprosil-PUR 3µm (New Objectives) and Trap column Acquity uPLC M-Class Symmetry C-18 column 100Å, 5µm, 2G 180µm x 20mm (Waters). Flow rate was set at 300 nL/min with a 5µL sample injection. The LC gradients for nanoflows were composed of 0.1% formic acid/LC-MS grade water and 0.1% formic acid/acetonitrile. For microflow analysis, the analytical column was a YMC-Triart C18 column, 12nm, 3µm (0.3mm I.D. x 150mm ID), and trap column - YMC-Triart C18 column, 12nm, S-3µm (0.5mm I.D. x 5mm). Microflow rate was set at 5 µL/min with a 10µL sample injection. For both LC methods, data were collected using data dependent acquisition (DDA) mode on a TripleTOF® 6600 System (Sciex) with the following settings; TOF-MS Mass Range – 400 -1250 Da Accumulation speed 250 msec; Product ion Mass Range 100 – 1500 Da Accumulation speed 50 msec; MS/MS Type High Sensitivity. Run time per sample in nanoflow was 135 min whereas a run time per samples in micro flow was 87 min.

For differential expression analysis, sequential window acquisition of all theoretical mass spectra (SWATH-MS) (11) was used was used as previously described (45). For each sample 50µg of protein was reduced, alkylated and digested prior to lyophilisation. Reduction was performed using 5mM dithiothreitol and sodium deoxycholate 1%, followed by incubation for 30 minutes at 60°C. Alkylation was performed using 50mM iodoacetamide for 30 minutes in the dark at room temperature. Overnight digestion was performed with 1:50 trypsin:protein at 37°C, followed by acidification to 0.5% formic acid and DOC removed by centrifugation 12K x g at 10°C.

Samples were randomised into 8 batches to run on the mass spectrometer, each batch consisting of 12 samples. Samples were reconstituted in loading buffer (2% (v/v) acetonitrile, 0.1% (v/v) formic acid, 10 fmol/µL PepCalMix (Sciex UK), and standard iRT peptides (46)) and loaded onto the autosampler in batches of 12 samples for sequential SWATH-MS analysisSamples were analysed by SWATH-MS with a micro-flow LC-MS system comprising an Eksigent nanoLC 400 autosampler and an Eksigent nanoLC 425 pump coupled to a SCIEX 6600 Triple-TOF mass spectrometer with a DuoSpray Ion Source. The system was configured for a trap-elute elute analysis. Sample was injected onto a trap column (YMC- Triart C18; length: 5 mm; ID: 0.5 mm; particle size: 3 µm; pore size: 120 Å) with loading buffer mobile phase (10 µL/min, 3 min, 2% (v/v) acetonitrile, 0.1% (v/v) formic acid). Peptides were then eluted through an analytical column (YMC-Triart C18; length: 150 mm; ID: 0.3 mm; particle size: 3 µm; pore size: 120 Å; 30°C) with the required analytical gradient into the mass spectrometer source. The system was controlled by Analyst software v1.7.1 and Eksigent control software v4.2. For SWATH-MS analysis the mass spectrometer method comprised a total duty cycle of 2.59s with a single TOF MS1 scan (m/z 400-1250, 250 ms) followed by 100 SWATH-MS scans (m/z 100-1500, 20ms) with variable m/z isolation widths, collision energy and collision energy spread.

In addition to the sample injections, a blank injection of loading buffer after each SWATH-MS and an auto-calibration injection of pepCalMix every sixteenth injection (4 µL, 10 fmol/µL, pepCalMix - Sciex, 2% acetonitrile, 0.1% formic acid) were analysed to check for batch errors.

## Supplementary Table 1: Longlist of 144 candidate protein biomarkers

| Rat gene name | Rat protein name | Number of peptides detected (different charge states considered different) | Pattern |
| --- | --- | --- | --- |
| MYL1 | Myosin light chain 1/3, skeletal muscle isoform | 1 | s/d/d |
| Crp | C-reactive protein | 1 | s/s/d |
| Psmb8 | Proteasome subunit beta type-8 | 1 | d/s/s |
| Krt17 | Keratin, type I cytoskeletal 17 | 1 | i/d/s |
| Arl8b | ADP-ribosylation factor-like protein 8B | 1 | s/?/i |
| Rabep1 | Rab GTPase-binding effector protein 1 | 1 | i/d/s |
| Ush2a | Usherin | 1 | s/d/d |
| H2ac18 | Histone H2A type 2-A | 1 | D/D/S |
| S100a4 | Protein S100-A4 | 1 | s/s/i |
| Dlg1 | Disks large homolog 1 | 2 | s/d/i |
| Actg1 | Actin, cytoplasmic 2 | 2 | s/d/d |
| Apoa2 | Apolipoprotein A-II | 2 | d/s/i |
| Gstm7 | Glutathione S-transferase Mu 7 | 2 | s/s/d |
| As3mt | Arsenite methyltransferase | 2 | s/d/i |
| Cst3 | Cystatin-C | 2 | s/i/i |
| Sparc | SPARC | 2 | d/s/i |
| F13a1 | Coagulation factor XIII A chain | 2 | D/S/D |
| Prss1 | Anionic trypsin-1 | 2 | s/s/d |
| Bag5 | BAG family molecular chaperone regulator 5 | 2 | s/?/s |
| Slc9a3r2 | Na(+)/H(+) exchange regulatory cofactor NHE-RF2 | 2 | s/d/? |
| Hnrnpd | Heterogeneous nuclear ribonucleoprotein D0 | 2 | s/?/s |
| Rhot2 | Mitochondrial Rho GTPase 2 | 2 | s/s/d |
| Prpsap2 | Phosphoribosyl pyrophosphate synthase-associated protein 2 | 2 | S/S/I |
| Acot7 | Cytosolic acyl coenzyme A thioester hydrolase | 2 | s/s/? |
| Apex1 | DNA-(apurinic or apyrimidinic site) endonuclease | 2 | d/s/i |
| Stx12 | Syntaxin-12 | 2 | ?/d/S |
| Actg2 | Actin, gamma-enteric smooth muscle | 2 | D/D/D |
| Eif2s3 | Eukaryotic translation initiation factor 2 subunit 3, X-linked | 2 | ?/d/s |
| Faf1 | FAS-associated factor 1 | 2 | s/s/? |
| Tomm34 | Mitochondrial import receptor subunit TOM34 | 2 | s/s/i |
| Stx7 | Syntaxin-7 | 2 | d/d/s |
| Rpl24 | 60S ribosomal protein L24 | 2 | s/s/i |
| Prune1 | Exopolyphosphatase PRUNE1 | 2 | s/s/i |
| Igkc | Ig kappa chain C region, B allele | 2 | D/S/D |
| Nit1 | Deaminated glutathione amidase | 2 | s/d/i |
| Agt | Angiotensinogen | 3 | ?/d/s |
| Camk2d | Calcium/calmodulin-dependent protein kinase type II subunit delta | 3 | s/d/i |
| Ehd3 | EH domain-containing protein 3 | 3 | s/i/i |
| Capzb | F-actin-capping protein subunit beta | 3 | s/d/d |
| Clic1 | Chloride intracellular channel protein 1 | 3 | s/d/i |
| Atp1b3 | Sodium/potassium-transporting ATPase subunit beta-3 | 3 | s/d/s |
| Ddx39b | Spliceosome RNA helicase Ddx39b | 3 | s/s/i |
| Ago2 | Protein argonaute-2 | 3 | s/d/s |
| Akap1 | A-kinase anchor protein 1, mitochondrial | 3 | s/d/s |
| Aldh3a1 | Aldehyde dehydrogenase, dimeric NADP-preferring | 3 | s/d/s |
| Erp29 | Endoplasmic reticulum resident protein 29 | 3 | S/S/I |
| Sars1 | Serine--tRNA ligase, cytoplasmic | 3 | s/d/i |
| Uba3 | NEDD8-activating enzyme E1 catalytic subunit | 3 | s/?/s |
| Fam136a | Protein FAM136A | 3 | s/i/d |
| Maip1 | m-AAA protease-interacting protein 1, mitochondrial | 3 | i/s/i |
| Actn4 | Alpha-actinin-4 | 4 | s/s/d |
| Tmem38a | Trimeric intracellular cation channel type A | 4 | s/d/i |
| Sirt2 | NAD-dependent protein deacetylase sirtuin-2 | 4 | s/d/i |
| Gnb3 | Guanine nucleotide-binding protein G(I)/G(S)/G(T) subunit beta-3 | 4 | s/s/d |
| Hdlbp | Vigilin | 4 | s/s/i |
| Parva | Alpha-parvin | 4 | s/s/i |
| C4bpa | C4b-binding protein alpha chain | 4 | s/s/i |
| Flot2 | Flotillin-2 | 4 | ?/s/s |
| Eif3i | Eukaryotic translation initiation factor 3 subunit I | 4 | s/d/i |
| Mgll | Monoglyceride lipase | 4 | d/s/i |
| Adhfe1 | Hydroxyacid-oxoacid transhydrogenase, mitochondrial | 4 | s/s/? |
| Retsat | All-trans-retinol 13,14-reductase | 4 | d/?/d |
| Nae1 | NEDD8-activating enzyme E1 regulatory subunit | 4 | I/S/I |
| Acta1 | Actin, alpha skeletal muscle | 5 | s/d/d |
| Ttr | Transthyretin | 5 | D/S/D |
| Ap2m1 | AP-2 complex subunit mu | 5 | ?/s/s |
| Mtm1 | Myotubularin | 5 | i/d/s |
| Glul | Glutamine synthetase | 5 | D/D/S |
| Sirt5 | NAD-dependent protein deacylase sirtuin-5, mitochondrial | 5 | s/d/s |
| Ca3 | Carbonic anhydrase 3 | 5 | s/d/i |
| Crip2 | Cysteine-rich protein 2 | 5 | S/D/D |
| Cryl1 | Lambda-crystallin homolog | 5 | S/D/S |
| Mrpl37 | 39S ribosomal protein L37, mitochondrial | 5 | d/?/s |
| Prep | Prolyl endopeptidase | 5 | s/d/i |
| Scl12a7 | Solute carrier family 12 member 7 | 5 | d/i/d |
| Ctnnb1 | Catenin beta-1 | 6 | s/d/i |
| Gnb1 | Guanine nucleotide-binding protein G(I)/G(S)/G(T) subunit beta-1 | 6 | s/d/i |
| Tomm70 | Mitochondrial import receptor subunit TOM70 | 6 | S/D/S |
| Crym | Ketimine reductase mu-crystallin | 6 | s/s/i |
| Ppp1r12a | Protein phosphatase 1 regulatory subunit 12A | 6 | S/i/i |
| Cap1 | Adenylyl cyclase-associated protein 1 | 7 | ?/s/s |
| Mfn1 | Mitofusin-1 | 7 | s/d/s |
| Por | NADPH--cytochrome P450 reductase | 7 | ?/s/s |
| Qars1 | Glutamine--tRNA ligase | 7 | s/d/s |
| Ociad1 | OCIA domain-containing protein 1 | 7 | s/d/i |
| Ahsg | Alpha-2-HS-glycoprotein | 8 | s/d/d |
| Ap2a2 | AP-2 complex subunit alpha-2 | 8 | I/S/I |
| Hprt1 | Hypoxanthine-guanine phosphoribosyltransferase | 8 | s/s/i |
| Gars1 | Glycine--tRNA ligase | 8 | s/s/? |
| Igg-2a | Ig gamma-2A chain C region | 9 | D/D/D |
| Clu | Clusterin | 10 | ?/s/i |
| Ywhah | 14-3-3 protein eta | 10 | s/d/s |
| Ddx1 | ATP-dependent RNA helicase DDX1 | 10 | i/d/s |
| Hnrnpl | Heterogeneous nuclear ribonucleoprotein L | 10 | S/S/I |
| Cap2 | Adenylyl cyclase-associated protein 2 | 10 | s/d/i |
| Nlrx1 | NLR family member X1 | 10 | s/s/d |
| Slc4a1 | Band 3 anion transport protein | 10 | s/s/d |
| Tmod1 | Tropomodulin-1 | 11 | s/d/d |
| XDH | Xanthine dehydrogenase/oxidase | 11 | S/D/S |
| Hspb6 | Heat shock protein beta-6 | 11 | s/s/i |
| Serpina3l | Serine protease inhibitor A3L | 11 | s/s/d |
| Gsn | Gelsolin | 12 | s/d/d |
| Hrg | Histidine-rich glycoprotein | 12 | S/s/d |
| Idh1 | Isocitrate dehydrogenase [NADP] cytoplasmic | 12 | s/s/i |
| Klhl41 | Kelch-like protein 41 | 12 | s/d/i |
| Ywhag | 14-3-3 protein gamma | 12 | S/S/I |
| Pdk1 | [Pyruvate dehydrogenase (acetyl-transferring)] kinase isozyme 1, mitochondrial | 12 | i/i/? |
| F2 | Prothrombin | 13 | i/i/i |
| Pdlim1 | PDZ and LIM domain protein 1 | 13 | s/d/i |
| Gc | Vitamin D-binding protein | 13 | s/s/d |
| Klybl | Citramalyl-CoA lyase, mitochondrial | 13 | i/s/? |
| Kyat3 | Kynurenine--oxoglutarate transaminase 3 | 13 | s/d/d |
| Apoe | Apolipoprotein E | 14 | s/s/i |
| Hsd17b4 | Peroxisomal multifunctional enzyme type 2 | 14 | s/s/i |
| Serpina1 | Alpha-1-antiproteinase | 15 | s/s/d |
| Serpina3k | Serine protease inhibitor A3K | 15 | s/s/d |
| Cat | Catalase | 16 | s/s/i |
| Csrp3 | Cysteine and glycine-rich protein 3 | 17 | s/s/i |
| Apoa1 | Apolipoprotein A-I | 18 | s/s/i |
| Hsp90aa1 | Heat shock protein HSP 90-alpha | 18 | s/s/i |
| Hspb1 | Heat shock protein beta-1 | 18 | s/s/i |
| Fhl1 | Four and a half LIM domains protein 1 | 19 | s/d/i |
| Gda | Guanine deaminase | 19 | ?/s/s |
| MVP | Major vault protein | 20 | I/I/I |
| Tcp1 | T-complex protein 1 subunit alpha | 21 | d/d/i |
| Hibch | 3-hydroxyisobutyryl-CoA hydrolase, mitochondrial | 21 | i/d/s |
| Plg | Plasminogen | 22 | I/S/D |
| Pgm1 | Phosphoglucomutase-1 | 24 | s/i/i |
| Fhl2 | Four and a half LIM domains protein 2 | 24 | s/s/d |
| Hpx | Hemopexin | 26 | s/s/d |
| Actc1 | Actin, alpha cardiac muscle 1 | 28 | s/d/d |
| Opa1 | Dynamin-like 120 kDa protein, mitochondrial | 29 | d/d/s |
| Pdia3 | Protein disulfide-isomerase A3 | 29 | S/s/i |
| Myl3 | Myosin light chain 3 | 30 | D/D/D |
| Hk1 | Hexokinase-1 | 30 | s/d/i |
| A1i3 | Alpha-1-inhibitor 3 | 30 | s/d/i |
| Des | Desmin | 36 | d/d/d |
| Lrpprc | Leucine-rich PPR motif-containing protein, mitochondrial | 45 | ?/s/i |
| Tf | Serotransferrin | 57 | s/s/d |
| Myh7 | Myosin-7 | 65 | d/d/d |
| Dmd | Dystrophin | 67 | S/s/i |
| A1m | Alpha-1-macroglobulin | 71 | s/s/i |
| Ryr2 | Ryanodine receptor 2 | 90 | S/D/D |
| Myh6 | Myosin-6 | 139 | d/d/d |

## Supplementary Table 2: Differential protein expression at each time point

Supplementary Table 2: The 10 proteins showing greatest significant difference in expression at each time point

| **Group 1 (1 dose)** | | | | |
| --- | --- | --- | --- | --- |
| **logFC** | **P.Value** | **Accession** | **Protein names** | **Gene names** |
| -10.989 | 0.000 | Q2TA68 | Dynamin-like 120 kDa protein, mitochondrial | Opa1 |
| 3.688 | 0.001 | P09606 | Glutamine synthetase (GS) | Glul |
| -3.2 | 0.008 | Q8K1Q0 | Glycylpeptide N-tetradecanoyltransferase 1 | Nmt1 |
| 3.028 | 0.002 | Q9Z1A5 | NEDD8-activating enzyme E1 regulatory subunit | Nae1 |
| -2.861 | 0.006 | Q5RK27 | Solute carrier family 12 member 7 | Slc12a7 |
| -2.695 | 0.015 | Q68FR9 | Elongation factor 1-delta | Eef1d |
| -2.617 | 0.018 | P04638 | Apolipoprotein A-II | Apoa2 |
| -2.553 | 0.000 | P00388 | NADPH--cytochrome P450 reductase | Por |
| -2.479 | 0.001 | Q8K4F7 | m7GpppX diphosphatase | Dcps |
| -2.433 | 0.000 | P08289 | Alkaline phosphatase, tissue-nonspecific isozyme | Alpl |
| **Group 2 (2 doses)** | | | | |
| **logFC** | **P.Value** | **Accession** | **Protein names** | **Gene names** |
| -7.944 | 0.009 | Q2TA68 | Dynamin-like 120 kDa protein, mitochondrial | Opa1 |
| 4.707 | 0 | P61149 | Fibroblast growth factor 1 | Fgf1 |
| -3.558 | 0.041 | P16975 | SPARC (Basement-membrane protein 40) | Sparc |
| -3.371 | 0.018 | Q10743 | Disintegrin and metalloproteinase domain-containing protein 10 | Adam10 |
| 3.285 | 0.005 | Q8VHU4 | Elongator complex protein 1 | Elp1 |
| 2.819 | 0.008 | Q10758 | Keratin, type II cytoskeletal 8 | Krt8 |
| -2.722 | 0.017 | P04644 | 40S ribosomal protein S17 | Rps17 |
| -2.497 | 0.018 | P20762 | Ig gamma-2C chain C region | IGG2C |
| 2.453 | 0.013 | Q5XIE6 | 3-hydroxyisobutyryl-CoA hydrolase, | Hibch |
|  |  |  | mitochondrial |  |
| -2.053 | 0.016 | Q9ER24 | Ataxin-10 | Atxn10 |
| **Group 3 (3 doses)** | | | | |
| **logFC** | **P.Value** | **Accession** | **Protein names** | **Gene names** |
| -7.297 | 0.016 | Q2TA68 | Dynamin-like 120 kDa protein, mitochondrial | Opa1 |
| -3.474 | 0.005 | B0BNF1 | Septin-8 | Sep-08 |
| -3.201 | 0.038 | Q5QJC9 | BAG family molecular chaperone regulator 5 | Bag5 |
| -2.908 | 0.011 | Q7TQ94 | Deaminated glutathione amidase | Nit1 |
| -2.689 | 0.039 | Q66HA6 | ADP-ribosylation factor-like protein 8B | Arl8b |
| 2.673 | 0.004 | A1A5Q0 | Leiomodin-2 | Lmod2 |
| -2.377 | 0.044 | Q03626 | Murinoglobulin-1 | Mug1 |
| 2.345 | 0.01 | Q62736 | Non-muscle caldesmon | Cald1 |
| 2.133 | 0.049 | P13635 | Ceruloplasmin | Cp |
| -2.115 | 0.02 | Q99MI7 | NEDD8-activating enzyme E1 catalytic subunit | Uba3 |
| **Group 4 (5 doses)** | | | | |
| **logFC** | **P.Value** | **Accession** | **Protein names** | **Gene names** |
| -6.742 | 0.016 | P17209 | Myosin light chain 4 | Myl4 |
| -5.402 | 0.004 | P01161 | Natriuretic peptides A | Nppa |
| -4.632 | 0.002 | P14141 | Carbonic anhydrase 3 | Ca3 |
| -3.835 | 0.003 | Q75Q39 | Mitochondrial import receptor subunit TOM70 | Tomm70 |
| 3.747 | 0.025 | Q66HG9 | Mitochondrial antiviral-signaling protein | Mavs |
| -3.228 | 0.024 | P15800 | Laminin subunit beta-2 | Lamb2 |
| -2.959 | 0.026 | P09606 | Glutamine synthetase | Glul |
| -2.879 | 0.016 | Q62696 | Disks large homolog 1 | Dlg1 |
| -2.86 | 0.004 | P52481 | Adenylyl cyclase-associated protein 2 | Cap2 |
| -2.687 | 0.035 | Q07984 | Translocon-associated protein subunit delta | Ssr4 |
| **Group 5 (7 doses)** | | | | |
| **logFC** | **P.Value** | **Accession** | **Protein names** | **Gene names** |
| 3.913 | 0.001 | P11530 | Dystrophin | Dmd |
| 3.852 | 0.001 | Q63065 | Pyruvate dehydrogenase (acetyl-transferring) kinase isozyme 1, mitochondrial | Pdk1 |
| 3.818 | 0 | Q924K2 | FAS-associated factor 1 | Faf1 |
| -3.464 | 0.001 | P21139 | Alpha-mannosidase 2C1 | Man2c1 |
| 3.333 | 0.016 | Q7TQ94 | Deaminated glutathione amidase | Nit1 |
| -3.063 | 0.003 | Q9WTV0 | Prolactin regulatory element-binding protein | Preb |
| 2.969 | 0.026 | Q8VHT6 | Arsenite methyltransferase | As3mt |
| 2.916 | 0.029 | P19332 | Microtubule-associated protein tau | Mapt |
| 2.545 | 0.018 | Q63514 | C4b-binding protein alpha chain | C4bpa |
| 2.535 | 0.044 | P18484 | AP-2 complex subunit alpha-2 | Ap2a2 |
| **Group 6 (7 doses + 4 weeks off treatment)** | | | | |
| **logFC** | **P.Value** | **Accession** | **Protein names** | **Gene names** |
| 6.977 | 0.01 | Q4FZU2 | Keratin, type II cytoskeletal 6A | Krt6a |
| 6.963 | 0.001 | P16975 | SPARC (Basement-membrane protein 40) | Sparc |
| 5.032 | 0.001 | P04638 | Apolipoprotein A-II | Apoa2 |
| -4.547 | 0 | P20760 | Ig gamma-2A chain C region | Igg-2a |
| 3.788 | 0.002 | Q8R491 | EH domain-containing protein 3 | Ehd3 |
| 3.633 | 0.01 | P18484 | AP-2 complex subunit alpha-2 | Ap2a2 |
| 3.276 | 0.006 | Q4QQW3 | Hydroxyacid-oxoacid transhydrogenase, mitochondrial | Adhfe1 |
| 2.944 | 0 | P04639 | Apolipoprotein A-I | Apoa1 |
| 2.864 | 0.002 | Q62667 | Major vault protein (MVP) | Mvp |
| 2.794 | 0.047 | P04642 | L-lactate dehydrogenase A chain | Ldha |

## Supplementary Figure 1a-f: Principal component analysis at each time point


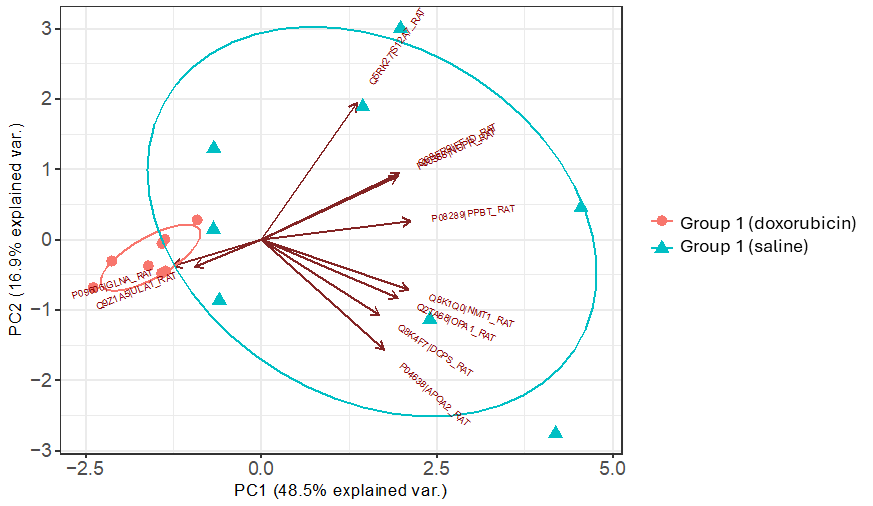

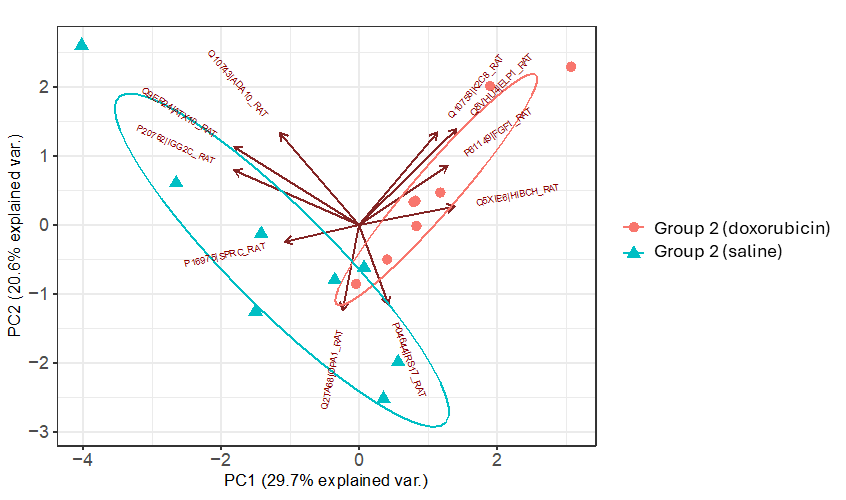


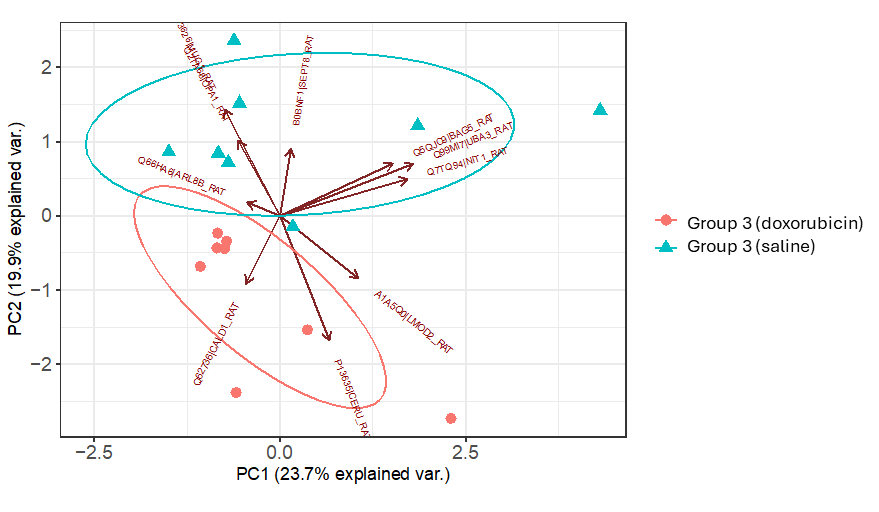


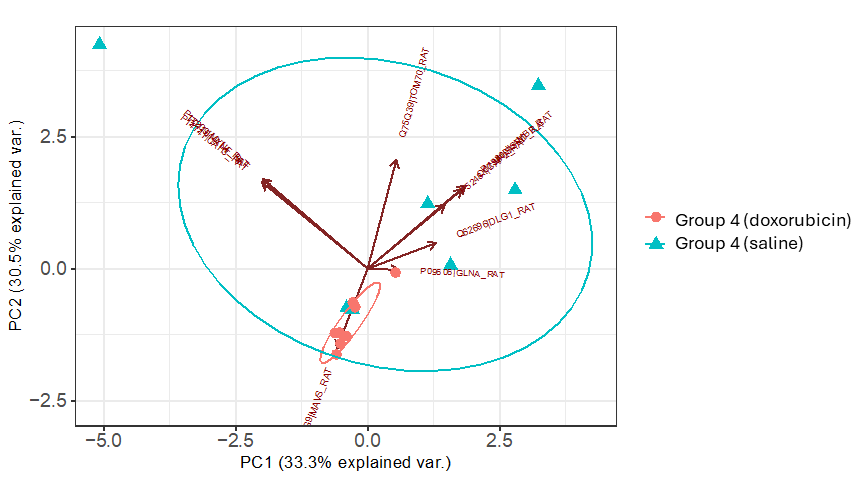


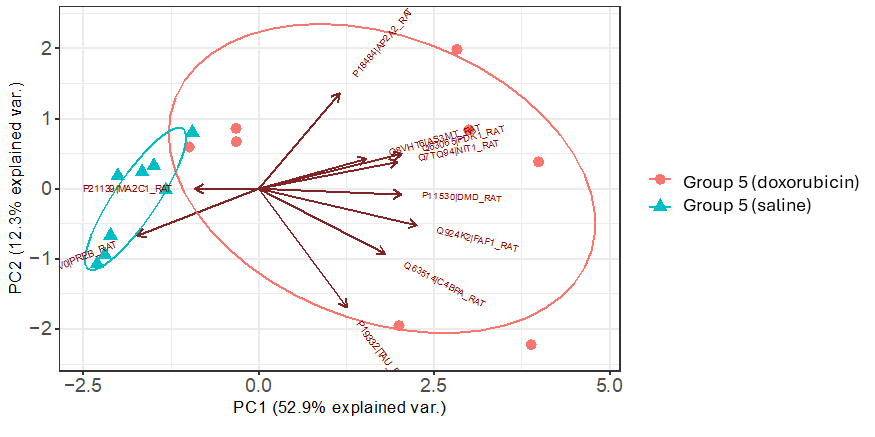


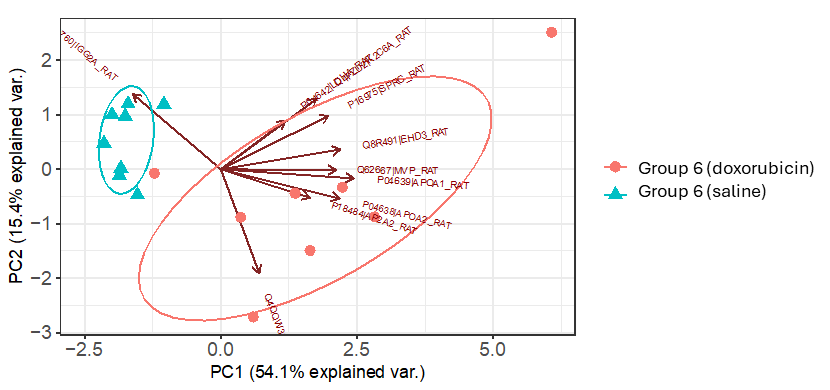


*Figures 1a-1f: Principal component analysis of proteins differentially expressed in each group (plots a to f corresponding to groups 1-6 respectively). The top 10 proteins showing significant difference at each timepoint are shown. PC1 and PC2 represent the first and second principal components, with explained variance (explained var.) shown on each plot.*

## Supplementary Table 3: Differential protein expression at sequential time points

Supplementary Table 3: The top 10 proteins showing a significant difference in expression between each time point in doxorubicin-treated rats

| **Week 1 to week 2** | | | | |
| --- | --- | --- | --- | --- |
| **logFC** | **P.Value** | **Accession** | **Protein names** | **Gene names** |
| -3.412 | 0.002 | P09606 | Glutamine synthetase | Glul |
| 2.976 | 0.012 | P18484 | AP-2 complex subunit alpha-2 | Ap2a2 |
| 2.851 | 0.002 | Q62667 | Major vault protein (MVP) | Mvp |
| 2.754 | 0 | P01015 | Angiotensinogen | Agt |
| 2.492 | 0.001 | P63326 | 40S ribosomal protein S10 | Rps10 |
| 2.447 | 0.027 | Q923M1 | Mitochondrial peptide methionine sulfoxide reductase | Msra |
| 2.374 | 0.001 | P81795 | Eukaryotic translation initiation factor 2 subunit 3, X-linked | Eif2s3 |
| 2.364 | 0.047 | Q62736 | Non-muscle caldesmon | Cald1 |
| 2.354 | 0.009 | Q5XIE6 | 3-hydroxyisobutyryl-CoA hydrolase, mitochondrial | Hibch |
| 2.281 | 0.004 | Q5XI72 | Eukaryotic translation initiation factor 4H | Eif4h |
| **Week 2 to week 3** | | | | |
| **logFC** | **P.Value** | **Accession** | **Protein names** | **Gene names** |
| -3.365 | 0.002 | P21139 | Alpha-mannosidase 2C1 | Man2c1 |
| -3.238 | 0.02 | P50475 | Alanine--tRNA ligase, cytoplasmic | Aars1 |
| 3.033 | 0 | Q9JJ31 | Cullin-5 | Cul5 |
| 2.922 | 0 | Q5RJP0 | Aldo-keto reductase family 1 member B7 | Akr1b7 |
| 2.666 | 0.022 | Q8R491 | EH domain-containing protein 3 | Ehd3 |
| -2.644 | 0 | P01015 | Angiotensinogen | Agt |
| -2.434 | 0.038 | Q63083 | Nucleobindin-1 | Nucb1 |
| -2.395 | 0.002 | P63326 | 40S ribosomal protein S10 | Rps10 |
| -2.332 | 0.004 | P81795 | Eukaryotic translation initiation factor 2 subunit 3, X-linked | Eif2s3 |
| -2.226 | 0.02 | Q01992 | Mitochondrial intermediate peptidase | Mipep |
| **Week 3 to week 5** | | | | |
| **logFC** | **P.Value** | **Accession** | **Protein names** | **Gene names** |
| -3.959 | 0.009 | Q6IG03 | Keratin, type II cytoskeletal 73 | Krt73 |
| 3.653 | 0.002 | P14841 | Cystatin-C | Cst3 |
| -2.708 | 0.014 | P11883 | Aldehyde dehydrogenase, dimeric NADP-preferring | Aldh3a1 |
| -2.282 | 0.05 | Q9JJ31 | Cullin-5 | Cul5 |
| -2.219 | 0.006 | Q9QZ81 | Protein argonaute-2 | Ago2 |
| 2.187 | 0.001 | Q6AXM7 | HBS1-like protein | Hbs1l |
| -2.072 | 0.016 | Q75Q39 | Mitochondrial import receptor subunit TOM70 | Tomm70 |
| -2.066 | 0.012 | B0BNA7 | Eukaryotic translation initiation factor 3 subunit I | Eif3i |
| 2.037 | 0.018 | Q6AXT0 | 39S ribosomal protein L37, mitochondrial | Mrpl37 |
| -1.933 | 0.022 | Q8VHE9 | All-trans-retinol 13,14-reductase | Retsat |
| **Week 5 to week 7** | | | | |
| **logFC** | **P.Value** | **Accession** | **Protein names** | **Gene names** |
| -3.34 | 0.004 | Q5U3Z3 | Isochorismatase domain-containing protein 2 | Isoc2 |
| -3.27 | 0.002 | Q4QQW3 | Hydroxyacid-oxoacid transhydrogenase, mitochondrial (HOT) | Adhfe1 |
| -3.12 | 0.002 | Q5RK27 | Solute carrier family 12 member 7 (Electroneutral potassium-chloride cotransporter 4) | Slc12a7 |
| 3.092 | 0.002 | Q62696 | Disks large homolog 1 (Synapse-associated protein 97) | Dlg1 |
| 2.987 | 0.029 | Q7TQ94 | Deaminated glutathione amidase (dGSH amidase) | Nit1 |
| 2.863 | 0.025 | P11530 | Dystrophin | Dmd |
| -2.812 | 0.004 | P22734 | Catechol O-methyltransferase | Comt |
| 2.73 | 0.001 | P50137 | Transketolase (TK) | Tkt |
| 2.677 | 0.002 | P14141 | Carbonic anhydrase 3 | Ca3 |
| 2.441 | 0.01 | Q9WU82 | Catenin beta-1 (Beta-catenin) | Ctnnb1 |
| **Week 7 to 4 weeks off** | | | | |
| **logFC** | **P.Value** | **Accession** | **Protein names** | **Gene names** |
| 5.17 | 0 | P16975 | SPARC (Basement-membrane protein 40) | Sparc |
| 3.484 | 0.029 | P04638 | Apolipoprotein A-II | Apoa2 |
| 3.463 | 0.042 | Q66HG9 | Mitochondrial antiviral-signaling protein (MAVS) | Mavs |
| 3.306 | 0 | P30427 | Plectin | Plec |
| -3.07 | 0.005 | Q924K2 | FAS-associated factor 1 | Faf1 |
| -2.945 | 0.011 | Q63065 | [Pyruvate dehydrogenase (acetyl-transferring)] kinase isozyme 1, mitochondrial | Pdk1 |
| 2.887 | 0.011 | Q4QQW3 | Hydroxyacid-oxoacid transhydrogenase, mitochondrial | Adhfe1 |
| 2.615 | 0.043 | Q8R491 | EH domain-containing protein 3 | Ehd3 |
| -2.143 | 0.004 | Q6AXM7 | HBS1-like protein | Hbs1l |
| 2.094 | 0.018 | Q63413 | Spliceosome RNA helicase Ddx39b | Ddx39b |

## Supplementary Table 4: Enriched pathways

In total, 365 enriched functional pathways were identified from the longlist of 144 proteins. When filtered by Observed proteins ≥4 AND Strength (Log10(observed / expected) ≥0.5 AND False discovery rate p<0.05, 186 enriched pathways remained, consisting of 129 unique proteins:

| **Enriched pathway and strength** | | **Observed proteins** |
| --- | --- | --- |
| **Muscle processes (n=44)** | | |
| Striated muscle contraction pathway | 1.58 | ACTN4, TMOD1, ACTC1, MYL1, DMD, ACTA1, DES, MYL3, MYH6, ACTG1 |
| Muscle filament sliding | 1.52 | TMOD1, ACTC1, MYL1, MYH7, DMD, ACTA1, DES, MYL3, MYH6 |
| Regulation of the force of heart contraction | 1.5 | CAMK2D, MYH7, RYR2, MYL3, MYH6, CSRP3 |
| Cardiac muscle hypertrophy | 1.39 | MYH7, RYR2, AGT, MYH6, CSRP3 |
| Striated muscle adaptation | 1.32 | MYH7, ACTA1, GSN, MYH6 |
| Actin-mediated cell contraction | 1.27 | TMOD1, ACTC1, MYL1, PARVA, CAMK2D, MYH7, DMD, RYR2, ACTA1, DES, MYL3, MYH6 |
| Cardiac muscle contraction | 1.21 | ACTC1, MYL1, CAMK2D, MYH7, DMD, RYR2, MYL3, MYH6, CSRP3 |
| Regulation of cardiac muscle cell apoptotic process | 1.21 | CAMK2D, AGT, HSPB6, SIRT5 |
| Myofibril assembly | 1.2 | TMOD1, KLHL41, ACTC1, ACTA1, MYH6, CSRP3, ACTG1 |
| Actin filament-based movement | 1.18 | ACTN4, TMOD1, ACTC1, MYL1, PARVA, CAMK2D, MYH7, DMD, RYR2, ACTA1, DES, MYL3, MYH6 |
| Hypertrophic cardiomyopathy | 1.15 | ACTC1, MYH7, DMD, RYR2, AGT, DES, MYL3, MYH6, ACTG1 |
| Sarcomere organization | 1.15 | KLHL41, MYH6, CSRP3, ACTG1 |
| Regulation of striated muscle contraction | 1.13 | TMEM38A, GSTM2, EHD3, CAMK2D, DLG1, MYH7, DMD, RYR2, MYL3 |
| Dilated cardiomyopathy | 1.13 | ACTC1, MYH7, DMD, RYR2, AGT, DES, MYL3, MYH6, ACTG1 |
| Striated muscle contraction | 1.1 | KLHL41, ACTC1, MYL1, CAMK2D, MYH7, DMD, RYR2, MYL3, MYH6, CSRP3 |
| Regulation of cardiac muscle contraction | 1.1 | TMEM38A, GSTM2, EHD3, CAMK2D, DLG1, DMD, RYR2 |
| Cardiac muscle cell development | 1.09 | ACTC1, AGT, MYH6, FHL2, CSRP3 |
| Ventricular cardiac muscle tissue morphogenesis | 1.07 | MYH7, RYR2, MYL3, MYH6 |
| Arrhythmogenic right ventricular cardiomyopathy | 1.06 | ACTN4, CTNNB1, DMD, RYR2, DES, ACTG1 |
| Striated muscle cell development | 1.03 | TMOD1, KLHL41, ACTC1, DMD, AGT, ACTA1, MYH6, FHL2, CSRP3, ACTG1 |
| Cardiac muscle tissue morphogenesis | 1.03 | ACTC1, MYH7, RYR2, MYL3, MYH6 |
| Muscle fiber development | 1.02 | KLHL41, DMD, ACTA1, MYH6 |
| Actomyosin structure organization | 1.01 | TMOD1, KLHL41, ACTC1, ACTA1, PDLIM1, MYH6, CSRP3, ACTG1 |
| Regulation of cardiac conduction | 1.01 | ATP1B3, EHD3, CAMK2D, RYR2, AGT |
| Muscle contraction | 0.96 | TMOD1, KLHL41, ACTC1, MYL1, CAMK2D, DLG1, MYH7, DMD, RYR2, AGT, ACTA1, DES, MYL3, MYH6, ACTG2, CSRP3 |
| Regulation of muscle contraction | 0.94 | TMEM38A, GSTM2, EHD3, CAMK2D, DLG1, MYH7, DMD, RYR2, MYL3, HSPB6 |
| Muscle system process | 0.93 | GSTM2, TMOD1, KLHL41, ACTC1, MYL1, CAMK2D, DLG1, MYH7, DMD, RYR2, AGT, ACTA1, GSN, DES, MYL3, MYH6, ACTG2, CSRP3 |
| Regulation of heart rate | 0.93 | CAMK2D, MYH7, DMD, RYR2, AGT, MYH6 |
| Myometrial relaxation and contraction pathways | 0.92 | GNB3, YWHAH, ACTC1, YWHAG, CAMK2D, RYR2, ACTA1, ACTG1, GNB1 |
| Regulation of heart contraction | 0.91 | TMEM38A, GSTM2, ATP1B3, EHD3, CAMK2D, DLG1, MYH7, DMD, RYR2, AGT, DES, MYL3, MYH6, CSRP3 |
| Adrenergic signaling in cardiomyocytes | 0.88 | ATP1B3, ACTC1, CAMK2D, MYH7, RYR2, AGT, MYL3, MYH6 |
| Regulation of muscle system process | 0.86 | TMEM38A, GSTM2, EHD3, CAMK2D, DLG1, MYH7, DMD, RYR2, AGT, MYL3, DDX39B, HSPB6 |
| Cardiac muscle tissue development | 0.85 | ACTC1, MYH7, RYR2, AGT, MYL3, MYH6, FHL2, CSRP3 |
| Cardiac chamber morphogenesis | 0.82 | PARVA, MYH7, RYR2, MYL3, MYH6, FHL2 |
| Muscle cell differentiation | 0.8 | TMOD1, KLHL41, ACTC1, CTNNB1, DMD, AGT, ACTA1, MYH6, FHL2, CSRP3, ACTG1 |
| Actin filament-based process | 0.74 | CAP2, ACTN4, TMOD1, KLHL41, ACTC1, MYL1, PARVA, CAMK2D, DLG1, MYH7, DMD, RYR2, ACTA1, PDLIM1, CAP1, GSN, DES, MYL3, TF, MYH6, CAPZB, CSRP3, ACTG1 |
| Actin filament organization | 0.74 | CAP2, ACTN4, TMOD1, ACTC1, DLG1, ACTA1, PDLIM1, CAP1, GSN, TF |
| Muscle tissue development | 0.72 | KLHL41, ACTC1, DLG1, MYH7, RYR2, AGT, ACTA1, MYL3, MYH6, FHL2, CSRP3 |
| Muscle structure development | 0.7 | TMOD1, KLHL41, ACTC1, PLG, CTNNB1, MYH7, DMD, RYR2, AGT, ACTA1, PDLIM1, FHL1, MYL3, MYH6, FHL2, CSRP3, ACTG1 |
| Striated muscle tissue development | 0.7 | KLHL41, ACTC1, MYH7, RYR2, AGT, ACTA1, MYL3, MYH6, FHL2, CSRP3 |
| Muscle organ development | 0.68 | KLHL41, ACTC1, MYH7, DMD, RYR2, ACTA1, FHL1, MYL3, MYH6, CSRP3 |
| Actin cytoskeleton organization | 0.64 | CAP2, ACTN4, TMOD1, KLHL41, ACTC1, PARVA, DLG1, ACTA1, PDLIM1, CAP1, GSN, TF, MYH6, CAPZB, CSRP3, ACTG1 |
| Regulation of actin filament-based process | 0.56 | HRG, APOA1, TMOD1, CAMK2D, DLG1, RYR2, GSN, CAPZB, CSRP3, ACTG1 |
| Heart development | 0.51 | SPARC, ACTC1, PARVA, CTNNB1, MYH7, RYR2, AGT, PDLIM1, MYL3, MYH6, FHL2, CSRP3 |
| **Metal ion processes (n=16)** | | |
| Regulation of cardiac muscle contraction by regulation of the release of sequestered calcium ion | 1.53 | TMEM38A, GSTM2, CAMK2D, DMD, RYR2 |
| Regulation of sodium ion transmembrane transport | 1.2 | YWHAH, ACTN4, ATP1B3, CAMK2D, DLG1, DMD, AGT |
| Regulation of sodium ion transmembrane transporter activity | 1.13 | YWHAH, ACTN4, CAMK2D, DMD, AGT |
| Regulation of release of sequestered calcium ion into cytosol | 1.03 | TMEM38A, GSTM2, F2, CAMK2D, DMD, RYR2 |
| Positive regulation of ion transmembrane transporter activity | 0.91 | GSTM2, ACTN4, ATP1B3, EHD3, DMD, RYR2 |
| Regulation of calcium ion transmembrane transporter activity | 0.88 | GSTM2, EHD3, CAMK2D, DMD, RYR2 |
| Calcium regulation in cardiac cells | 0.82 | GNB3, YWHAH, ATP1B3, YWHAG, CAMK2D, RYR2, GNB1 |
| Regulation of calcium ion transmembrane transport | 0.81 | TMEM38A, GSTM2, F2, EHD3, CAMK2D, DMD, RYR2 |
| Positive regulation of ion transport | 0.77 | APOA1, GSTM2, APOE, ACTN4, ATP1B3, F2, EHD3, DLG1, DMD, RYR2, AGT, FHL1 |
| Regulation of ion transmembrane transporter activity | 0.77 | GSTM2, YWHAH, ACTN4, ATP1B3, EHD3, CAMK2D, DLG1, DMD, RYR2, AGT, FHL1 |
| Regulation of metal ion transport | 0.73 | TMEM38A, GSTM2, YWHAH, ACTN4, ATP1B3, F2, EHD3, CAMK2D, CTNNB1, DLG1, DMD, RYR2, AGT, FHL1, TF |
| Regulation of calcium ion transport | 0.69 | TMEM38A, GSTM2, F2, EHD3, CAMK2D, CTNNB1, DMD, RYR2, AGT |
| Regulation of ion transmembrane transport | 0.62 | TMEM38A, GSTM2, YWHAH, ACTN4, ATP1B3, F2, EHD3, CAMK2D, DLG1, DMD, RYR2, AGT, CLIC1, FHL1 |
| Regulation of ion transport | 0.56 | TMEM38A, APOA1, GSTM2, YWHAH, APOE, ACTN4, ATP1B3, F2, EHD3, CAMK2D, CTNNB1, DLG1, DMD, RYR2, AGT, CLIC1, FHL1, TF |
| Cellular metal ion homeostasis | 0.51 | TMEM38A, GSTM2, APOE, HPX, ATP1B3, C2orf47, F2, CAMK2D, RYR2, AGT, TF, CSRP3, GNB1 |
| Metal ion homeostasis | 0.5 | GSTM2, APOE, SLC12A7, HPX, ATP1B3, C2orf47, F2, CAMK2D, RYR2, AGT, TF, CSRP3, GNB1, GNB1 |
| **Haemostasis and angiogenesis (n=14)** | | |
| Platelet degranulation | 1.12 | SPARC, HRG, APOA1, ACTN4, F13A1, PLG, CLU, CAP1, GIG25, TF, AHSG, SERPINA1 |
| Negative regulation of blood coagulation | 1.08 | HRG, APOE, F2, PLG |
| Complement and coagulation cascades | 1.01 | F13A1, F2, PLG, CLU, C4BPA, SERPINA1 |
| Positive regulation of wound healing | 0.98 | HRG, F2, PLG, ACTG1 |
| Platelet activation, signaling and aggregation | 0.94 | GNB3, SPARC, HRG, APOA1, ACTN4, F13A1, MGLL, F2, PLG, CLU, CAP1, GIG25, TF, AHSG, SERPINA1, GNB1 |
| Negative regulation of blood vessel morphogenesis | 0.85 | SPARC, HRG, SARS, CTNNB1, AGT, XDH |
| Platelet activation | 0.8 | HRG, HSPB1, F2, CLIC1, ACTG1, GNB1 |
| Blood coagulation | 0.78 | HRG, HSPB1, SLC4A1, F13A1, F2, PLG, EHD3, CLIC1, CAPZB, SERPINA1, ACTG1, AKAP1, GNB1 |
| Hemostasis | 0.69 | GNB3, SPARC, HRG, APOA1, ACTN4, F13A1, MGLL, ATP1B3, F2, PLG, CLU, EHD3, CAP1, GIG25, TF, AHSG, CAPZB, SERPINA1, MFN1, AKAP1, GNB1 |
| Blood circulation | 0.67 | GNB3, APOE, CRP, ACTC1, MYL1, CAMK2D, MYH7, DMD, RYR2, AGT, MYL3, MYH6, CSRP3 |
| Wound healing | 0.65 | SPARC, HRG, HSPB1, SLC4A1, F13A1, F2, PLG, EHD3, CLIC1, CAPZB, SERPINA1, ACTG1, AKAP1, GNB1 |
| Response to wounding | 0.63 | SPARC, HRG, APOA1, HSPB1, SLC4A1, F13A1, F2, PLG, EHD3, CLIC1, CST3, CAPZB, SERPINA1, ACTG1, AKAP1, GNB1 |
| Regulation of vasculature development | 0.62 | AGO2, SPARC, HRG, SARS, HSPB1, GLUL, CTNNB1, AGT, XDH, HSPB6 |
| Regulation of angiogenesis | 0.62 | AGO2, SPARC, HRG, SARS, HSPB1, GLUL, CTNNB1, AGT, HSPB6 |
| **Lipid processes (n=12)** | | |
| Positive regulation of cholesterol esterification | 1.8 | APOA1, APOE, AGT, APOA2 |
| High-density lipoprotein particle clearance | 1.75 | APOA1, APOE, APOA2, HDLBP |
| Reverse cholesterol transport | 1.52 | APOA1, APOE, CLU, APOA2 |
| Cholesterol efflux | 1.39 | APOA1, APOE, APOA2, STX12 |
| Plasma lipoprotein clearance | 1.33 | APOA1, APOE, AP2M1, AP2A2, HDLBP |
| Plasma lipoprotein particle clearance | 1.32 | APOA1, APOE, AP2M1, AP2A2, APOA2, HDLBP |
| Plasma lipoprotein particle remodeling | 1.27 | APOA1, APOE, AGT, APOA2 |
| Regulation of plasma lipoprotein particle levels | 1.12 | APOA1, APOE, AP2M1, AP2A2, AGT, APOA2, HDLBP |
| Cholesterol transport | 1.12 | APOA1, APOE, CLU, APOA2, STX12 |
| Plasma lipoprotein assembly, remodeling, and clearance | 1.08 | APOA1, APOE, AP2M1, AP2A2, APOA2, HDLBP |
| Positive regulation of lipid metabolic process | 0.74 | APOA1, APOE, F2, AGT, APOA2, POR |
| Regulation of lipid metabolic process | 0.56 | GNB3, APOA1, APOE, F2, AGT, APOA2, PDK1, GIG25, FHL2, IDH1, POR |
| **Inflammation and immunity (n=9)** | | |
| Acute-phase response | 1.2 | CRP, F2, GIG25, AHSG, SERPINA1 |
| Acute inflammatory response | 1.06 | CRP, F2, APOA2, GIG25, AHSG, SERPINA1 |
| Leukocyte mediated immunity | 0.64 | TTR, CAT, CRP, HPRT1, F2, CLU, AP2A2, HSP90AA1, DLG1, C4BPA, PGM1, CAP1, GSN, GIG25, MVP, CST3, IDH1, AHSG, SERPINA1, PRSS3P2 |
| Neutrophil mediated immunity | 0.63 | TTR, CAT, F2, AP2A2, HSP90AA1, PGM1, CAP1, GSN, GIG25, MVP, CST3, IDH1, AHSG, SERPINA1, PRSS3P2 |
| Neutrophil degranulation | 0.62 | TTR, CAT, AP2A2, HSP90AA1, PGM1, CAP1, GSN, GIG25, MVP, CST3, IDH1, AHSG, SERPINA1, PRSS3P2 |
| Myeloid leukocyte activation | 0.56 | TTR, CAT, CLU, AP2A2, HSP90AA1, PGM1, CAP1, GSN, GIG25, MVP, CST3, IDH1, AHSG, SERPINA1, PRSS3P2 |
| Regulation of immune effector process | 0.53 | APOA1, HPX, F2, CLU, C4BPA, STX7, APOA2, DDX1, NLRX1, HK1 |
| Innate Immune System | 0.5 | TTR, CAT, CRP, F2, CLU, AP2A2, HSP90AA1, CTNNB1, UBA3, C4BPA, PGM1, CAP1, GSN, PSMB8, GIG25, MVP, CST3, NLRX1, IDH1, AHSG, SERPINA1, ACTG1, PRSS3P2 |
| Immune effector process | 0.5 | TTR, CAT, CRP, HPRT1, F2, CLU, AP2A2, HSP90AA1, DLG1, C4BPA, PGM1, CAP1, GSN, DDX1, GIG25, MVP, CST3, IDH1, AHSG, SERPINA1, ACTG1, PRSS3P2, |
| **Mitochondrial processes (n=5)** | | |
| Mitochondrion localization | 1.18 | LRPPRC, RHOT2, OPA1, MTM1, MFN1 |
| Establishment of protein localization to mitochondrion | 0.98 | TOMM70A, C2orf47, HSP90AA1, TOMM34, HK1 |
| Mitochondrial membrane organization | 0.86 | YWHAH, C2orf47, YWHAG, RHOT2, HSP90AA1, OPA1, MFN1 |
| Mitochondrial transport | 0.7 | YWHAH, TOMM70A, C2orf47, YWHAG, RHOT2, HSP90AA1, OPA1, TOMM34 |
| Mitochondrion organization | 0.57 | YWHAH, TOMM70A, C2orf47, YWHAG, CLU, RHOT2, HSP90AA1, OPA1, MTM1, TOMM34, MFN1, SIRT5 |
| **Other metabolic processes (n=20)** | | |
| Positive regulation of steroid metabolic process | 1.39 | APOA1, APOE, AGT, APOA2, POR |
| Vitamin B12 metabolism | 1.23 | APOA1, APOE, CRP, F2, PLG, GIG25 |
| Retinoid metabolism and transport | 1.2 | APOA1, TTR, APOE, RETSAT, APOA2 |
| Guanosine-containing compound metabolic process | 1.16 | HPRT1, DLG1, OPA1, MFN1 |
| Folate metabolism | 1.09 | APOA1, CAT, CRP, F2, PLG, GIG25 |
| Regulation of steroid metabolic process | 0.84 | GNB3, APOA1, APOE, AGT, APOA2, POR |
| Nucleoside metabolic process | 0.83 | PRPSAP2, HPRT1, DLG1, OPA1, MFN1 |
| Secondary alcohol metabolic process | 0.81 | APOA1, CAT, APOE, APOA2, HDLBP, IDH1 |
| Regulation of reactive oxygen species metabolic process | 0.78 | SIRT2, CRP, F2, CLU, HSP90AA1, AGT, XDH, SIRT5 |
| Positive regulation of cellular amide metabolic process | 0.78 | HNRNPL, APOE, EIF2S3, KRT17, HNRNPD, CLU, DDX39B |
| Purine-containing compound metabolic process | 0.67 | TTR, GDA, PRPSAP2, HPRT1, DLG1, OPA1, PGM1, ACOT7, XDH, HK1, MFN1, HSD17B4 |
| Purine nucleotide metabolic process | 0.67 | GDA, PRPSAP2, HPRT1, DLG1, OPA1, PGM1, ACOT7, XDH, HK1, MFN1, HSD17B4 |
| Steroid metabolic process | 0.64 | APOA1, CAT, YWHAH, APOE, APOA2, HDLBP, HSD17B4, GC |
| Cellular amino acid metabolic process | 0.61 | CRYM, SARS, CCBL2, QARS, GLUL, HIBCH, GARS, ADHFE1 |
| Regulation of cellular amide metabolic process | 0.6 | AGO2, HNRNPL, HSPB1, APOE, EIF2S3, LRPPRC, KRT17, HNRNPD, CLU, EIF3I, DDX1, PDK1, DDX39B |
| Nucleotide metabolic process | 0.6 | GDA, PRPSAP2, HPRT1, DLG1, OPA1, PGM1, ACOT7, XDH, GARS, HK1, MFN1, HSD17B4 |
| Ribose phosphate metabolic process | 0.59 | PRPSAP2, HPRT1, DLG1, OPA1, PGM1, ACOT7, HK1, MFN1, HSD17B4 |
| Nucleobase-containing small molecule metabolic process | 0.56 | TTR, GDA, PRPSAP2, HPRT1, DLG1, OPA1, PGM1, ACOT7, XDH, GARS, HK1, MFN1, HSD17B4 |
| Organic hydroxy compound metabolic process | 0.56 | CRYM, APOA1, TTR, CAT, APOE, RETSAT, HPRT1, APOA2, HDLBP, IDH1, HSD17B4, GC |
| Positive regulation of reactive oxygen species metabolic process | 0.91 | CRP, F2, CLU, HSP90AA1, AGT, XDH |
| **Other processes (n=67)** | | |
| Purine nucleobase metabolic process | 1.41 | TTR, GDA, HPRT1, XDH |
| Disruption of postsynaptic signaling by CNV | 1.25 | YWHAG, CAMK2D, DLG1, RYR2 |
| Binding and Uptake of Ligands by Scavenger Receptors | 1.23 | SPARC, APOA1, APOE, HPX, HSP90AA1 |
| Positive regulation of telomerase activity | 1.19 | HNRNPD, TCP1, HSP90AA1, CTNNB1 |
| Mechanoregulation and pathology of YAP/TAZ via Hippo and non-Hippo mechanisms | 1.18 | ACTC1, CTNNB1, ACTA1, ACTG2, ACTG1 |
| Fas ligand pathway and stress induction of heat shock proteins | 1.13 | HSPB1, ACTA1, FAF1, ACTG1 |
| Purine nucleotide catabolic process | 1.12 | GDA, HPRT1, ACOT7, XDH |
| Selenium micronutrient network | 1.06 | APOA1, CAT, CRP, F2, PLG, XDH, GIG25 |
| Regulation of Insulin-like Growth Factor (IGF) transport and uptake by Insulin-like Growth Factor Binding Proteins (IGFBPs) | 1.01 | APOA1, APOE, F2, PLG, APOA2, CST3, TF, AHSG, SERPINA1 |
| Positive regulation of dna biosynthetic process | 1 | HNRNPD, TCP1, HSP90AA1, CTNNB1, DDX39B |
| Post-translational protein phosphorylation | 0.96 | APOA1, APOE, APOA2, CST3, TF, AHSG, SERPINA1 |
| Regulation of oxidoreductase activity | 0.95 | APOE, SLC4A1, HSP90AA1, AGT, PDK1, POR, SIRT5 |
| Positive regulation of reactive oxygen species metabolic process | 0.91 | CRP, F2, CLU, HSP90AA1, AGT, XDH |
| Positive regulation of cation transmembrane transport | 0.89 | GSTM2, ACTN4, ATP1B3, F2, EHD3, DMD, RYR2, AGT |
| Interaction with symbiont | 0.88 | HRG, APOE, CRP, F2, PLG |
| Tissue migration | 0.87 | APOA1, ACTC1, ACTA1, ACTG2, PRSS3P2 |
| Protein stabilization | 0.83 | APOA1, ATP1B3, CLU, TCP1, HSP90AA1, BAG5, APOA2, STX12, FLOT2 |
| Protein folding | 0.82 | GNB3, HSPB1, ERP29, PDIA3, CLU, TCP1, HSP90AA1, BAG5, HSPB6, GNB1 |
| Non-canonical wnt signaling pathway | 0.81 | AGO2, AP2M1, AP2A2, CTNNB1, PSMB8, GNB1 |
| Receptor-mediated endocytosis | 0.79 | SPARC, APOA1, APOE, HPX, AP2M1, AP2A2, HSP90AA1, CAP1 |
| Regulation of transporter activity | 0.78 | GSTM2, YWHAH, ACTN4, ATP1B3, EHD3, CAMK2D, DLG1, DMD, RYR2, AGT, APOA2, FHL1 |
| Regulation of endothelial cell migration | 0.78 | SPARC, HRG, HSPB1, APOE, GLUL, PLG, AGT |
| Positive regulation of apoptotic signaling pathway | 0.74 | YWHAH, PDIA3, YWHAG, CLU, AGT, GSN, FAF1 |
| Transport along microtubule | 0.74 | HSPB1, ARL8B, LRPPRC, RHOT2, OPA1, FLOT2 |
| Regulation of cation transmembrane transport | 0.73 | TMEM38A, GSTM2, YWHAH, ACTN4, ATP1B3, F2, EHD3, CAMK2D, DLG1, DMD, RYR2, AGT, FHL1 |
| Response to purine-containing compound | 0.73 | TMEM38A, APEX1, SPARC, GSTM2, RYR2, ALDH3A1 |
| Supramolecular fiber organization | 0.72 | CAP2, ACTN4, TMOD1, KLHL41, ACTC1, KRT17, DLG1, ACTA1, MTM1, PDLIM1, CAP1, GSN, DES, CST3, TF, MYH6, CSRP3, ACTG1 |
| Regulation of protein stability | 0.72 | APOA1, ATP1B3, CLU, TCP1, HSP90AA1, BAG5, APOA2, GSN, STX12, FLOT2, TF |
| Ribonucleoprotein complex assembly | 0.72 | AGO2, EIF2S3, HSP90AA1, EIF3I, DDX1, RPL24, DDX39B |
| Cytoskeleton-dependent intracellular transport | 0.7 | HSPB1, ACTN4, ARL8B, LRPPRC, RHOT2, OPA1, FLOT2 |
| Regulation of protein localization to membrane | 0.7 | YWHAH, AP2M1, YWHAG, DLG1, STX7, GSN, CSRP3 |
| Regulation of system process | 0.68 | TMEM38A, APOA1, GSTM2, APOE, MGLL, ATP1B3, EHD3, CAMK2D, DLG1, MYH7, DMD, RYR2, AGT, APOA2, DES, MYL3, DDX39B, MYH6, CSRP3, HSPB6 |
| Response to alcohol | 0.68 | SPARC, CAT, CA3, ACTC1, HNRNPD, CTNNB1, GSN, GNB1 |
| Response to hypoxia | 0.66 | CAT, SIRT2, ACTN4, OPA1, RYR2, PDLIM1, PSMB8, PDK1, CST3, ALDH3A1, GNB1 |
| Regulated exocytosis | 0.65 | SPARC, HRG, APOA1, TTR, CAT, ACTN4, F13A1, PLG, CLU, AP2A2, HSP90AA1, PGM1, CAP1, GSN, GIG25, MVP, CST3, TF, IDH1, AHSG, SERPINA1, PRSS3P2, |
| Regulation of apoptotic signaling pathway | 0.65 | HSPB1, YWHAH, ERP29, PDIA3, YWHAG, QARS, CLU, BAG5, CTNNB1, OPA1, AGT, GSN, FAF1 |
| Tissue homeostasis | 0.65 | HSPB1, USH2A, CTNNB1, DMD, GIG25, TF, ACTG1 |
| Membrane organization | 0.63 | APOA1, YWHAH, APOE, SLC4A1, AP2M1, C2orf47, YWHAG, CLU, RHOT2, AP2A2, HSP90AA1, DLG1, OPA1, STX7, APOA2, GSN, STX12, FLOT2, TF, SERPINA1, MFN1, CSRP3, RABEP1 |
| Endocytosis | 0.63 | SPARC, APOA1, APOE, HPX, AP2M1, EHD3, AP2A2, HSP90AA1, CAP1, GSN, AHSG, RABEP1, ACTG1 |
| Post-translational protein modification | 0.63 | APOA1, APOE, KLHL41, NAE1, UBA3, APOA2, PSMB8, CST3, TF, AHSG, SERPINA1 |
| Regulation of body fluid levels | 0.62 | HRG, HSPB1, APOE, SLC4A1, F13A1, F2, PLG, EHD3, CLIC1, XDH, CAPZB, SERPINA1, ACTG1, AKAP1, GNB1 |
| Anatomical structure homeostasis | 0.61 | APEX1, HSPB1, USH2A, PLG, HSP90AA1, CTNNB1, DMD, MTM1, GIG25, TF, ACTG1 |
| Negative regulation of proteolysis | 0.6 | HRG, F2, BAG5, AGT, MTM1, GIG25, CST3, AHSG, SERPINA1, POR |
| Establishment of protein localization to organelle | 0.59 | CAT, TOMM70A, C2orf47, CLU, HSP90AA1, RYR2, AGT, TOMM34, RPL24, IDH1, HK1, HSD17B4 |
| Cellular homeostasis | 0.58 | TMEM38A, APEX1, GNB3, GSTM2, APOE, SLC4A1, SLC12A7, HPX, ATP1B3, C2orf47, PDIA3, F2, PLG, RHOT2, CAMK2D, OPA1, DMD, RYR2, AGT, MTM1, TF, HK1, CSRP3 |
| Response to extracellular stimulus | 0.58 | HNRNPL, SPARC, APOA1, CAT, SIRT2, APOE, GLUL, ACTA1, GSN, CST3, ALDH3A1, POR, SIRT5 |
| Response to nutrient levels | 0.58 | HNRNPL, SPARC, APOA1, CAT, SIRT2, APOE, GLUL, GSN, CST3, ALDH3A1, POR, SIRT5 |
| Negative regulation of phosphorylation | 0.58 | HSPB1, SIRT2, APOE, SLC4A1, YWHAG, QARS, DLG1, DMD, AGT, XDH, MVP, AHSG |
| Negative regulation of intracellular signal transduction | 0.56 | GSTM2, HSPB1, APOE, QARS, CLU, BAG5, DLG1, OPA1, AGT, MTM1, XDH, FHL2, NLRX1 |
| VEGFA-VEGFR2 signaling pathway | 0.56 | SARS, HSPB1, TMOD1, HSP90AA1, CTNNB1, CLIC1, FAF1, FHL2, CAPZB, CRIP2, ACTG1 |
| Transport of small molecules | 0.55 | GNB3, APOA1, APOE, EIF2S3, SLC4A1, SLC12A7, ATP1B3, AP2M1, C2orf47, AP2A2, CAMK2D, RYR2, APOA2, PSMB8, HDLBP, TF, AKAP1, GNB1 |
| Regulation of membrane potential | 0.55 | YWHAH, ATP1B3, EHD3, CAMK2D, DLG1, DMD, RYR2, AGT, CLIC1, FHL1, MFN1 |
| Response to oxidative stress | 0.55 | APEX1, CAT, SIRT2, APOE, CA3, HNRNPD, PDLIM1, PDK1, CST3, IDH1 |
| Regulation of translation | 0.55 | AGO2, HNRNPL, HSPB1, EIF2S3, LRPPRC, KRT17, HNRNPD, EIF3I, DDX1, DDX39B |
| Protein targeting | 0.55 | CAT, TOMM70A, YWHAG, CLU, HSP90AA1, TOMM34, RPL24, IDH1, HSD17B4 |
| Secretion by cell | 0.54 | SPARC, HRG, APOA1, TTR, CAT, ACTN4, ERP29, F13A1, PLG, CLU, AP2A2, HSP90AA1, AGT, PGM1, CAP1, GSN, GIG25, MVP, CST3, TF, IDH1, AHSG, SERPINA1 |
| Regulation of protein-containing complex assembly | 0.54 | HRG, SIRT2, APOE, TMOD1, PRUNE, CLU, HSP90AA1, DLG1, GSN, FAF1, CAPZB |
| Export from cell | 0.53 | SPARC, HRG, APOA1, TTR, CAT, ACTN4, ERP29, F13A1, ATP1B3, PLG, CLU, AP2A2, HSP90AA1, AGT, PGM1, CAP1, GSN, GIG25, MVP, CST3, TF, IDH1, AHSG |
| Negative regulation of protein phosphorylation | 0.53 | HSPB1, SIRT2, APOE, YWHAG, QARS, DLG1, DMD, AGT, XDH, MVP |
| Regulation of endopeptidase activity | 0.53 | HRG, AGT, GSN, PSMB8, XDH, GIG25, CST3, AHSG, SERPINA1, POR |
| Negative regulation of catalytic activity | 0.52 | HRG, APOA1, HSPB1, APOE, SLC4A1, PRPSAP2, YWHAG, QARS, BAG5, AGT, APOA2, PDK1, GIG25, MVP, CST3, PPP1R12A, AHSG, SERPINA1, POR |
| Cellular response to organonitrogen compound | 0.52 | TMEM38A, APEX1, GSTM2, SIRT2, YWHAG, HNRNPD, CTNNB1, OPA1, RYR2, AGT, ACTA1, POR, CSRP3, GNB1 |
| Organelle localization | 0.52 | ACTN4, ARL8B, LRPPRC, YWHAG, RHOT2, HSP90AA1, CTNNB1, DLG1, OPA1, STX7, MTM1, STX12, SERPINA1, MFN1 |
| Small molecule catabolic process | 0.52 | CRYM, APOE, CRYL1, HPRT1, GLUL, HIBCH, PGM1, ADHFE1, HK1, HSD17B4 |
| Secretion | 0.51 | SPARC, HRG, APOA1, TTR, CAT, ACTN4, ERP29, F13A1, PLG, CLU, AP2A2, HSP90AA1, AGT, PGM1, CAP1, GSN, XDH, GIG25, MVP, CST3, TF, IDH1, AHSG |
| Response to organic cyclic compound | 0.51 | TMEM38A, APEX1, SPARC, CAT, GSTM2, YWHAH, SIRT2, HNRNPD, CTNNB1, OPA1, RYR2, AGT, ACTA1, APOA2, GSN, DDX1, CST3, FHL2, IDH1, ALDH3A1, GNB1 |
| Cell activation | 0.5 | HRG, TTR, CAT, HSPB1, HPRT1, F2, CLU, AP2A2, HSP90AA1, CTNNB1, DLG1, PGM1, CAP1, GSN, CLIC1, GIG25, MVP, CST3, IDH1, AHSG, SERPINA1, ACTG1, GNB1 |
